# Strong Affinity of Triazolium-Appended Dipyrromethenes (TADs) for BF_4_^−^

**DOI:** 10.3390/molecules25194555

**Published:** 2020-10-05

**Authors:** Charles Guérin, Zhan Zhang, Ludivine Jean-Gérard, Stephan Steinmann, Carine Michel, Bruno Andrioletti

**Affiliations:** 1 ICBMS-UMR 5246, Université de Lyon, Université Claude Bernard Lyon 1, Campus Lyon-Tech la Doua, Bâtiment Lederer, 1, Rue Victor Grignard, 69622 Villeurbanne, France; charles.guerin4@gmail.com (C.G.); zhanzhang1006@sina.com (Z.Z.); ludivine.jean-gerard@univ-lyon1.fr (L.J.-G.); 2 Laboratoire de Chimie, CNRS UMR 5182, Université de Lyon, ENS de Lyon, Site Monod, Bâtiment M6, 69364 Lyon, France; stephan.steinmann@ens-lyon.fr

**Keywords:** anions, host-guest systems, N ligands, triazolium, boron tetrafluoride

## Abstract

Because BF_4_^−^ is a labile, non- or weakly coordinating anion, it is generally chosen by chemists who do not want the anion to interfere with the associated cation. Herein, we demonstrate that BF_4_^−^ actually strongly binds to triazole-appended dipyrromethenes (TADs). In particular, HETCOR NMR experiments and DFT calculations were used to rationalize the results observed with anion titrations. Hence, special care should be taken when considering that BF_4_^−^ is innocent.

## 1. Introduction

Anions play major roles in various areas including industry (e.g., radioactive wastes), environment (e.g., nitrate related pollution), or biological systems (anion transport and sensing). Accordingly, the development of robust and selective systems is of prime interest. While studying anion recognition by positively charged hosts, researchers usually use fluorinated anions such as BF_4_^−^ and PF_6_^−^ as the counter anions [1,2,3,4], because these weakly coordinating anions do not compete with the coordination of more basic anions such as halides, carboxylates, nitrates, and so on. However, some rare examples are found in the literature in which these supposedly “innocent” fluorinated anions are involved in the assembly of cage complexes, [5,6,7,8,9] suggesting that they should not be considered as totally innocent.

**Figure 1:**
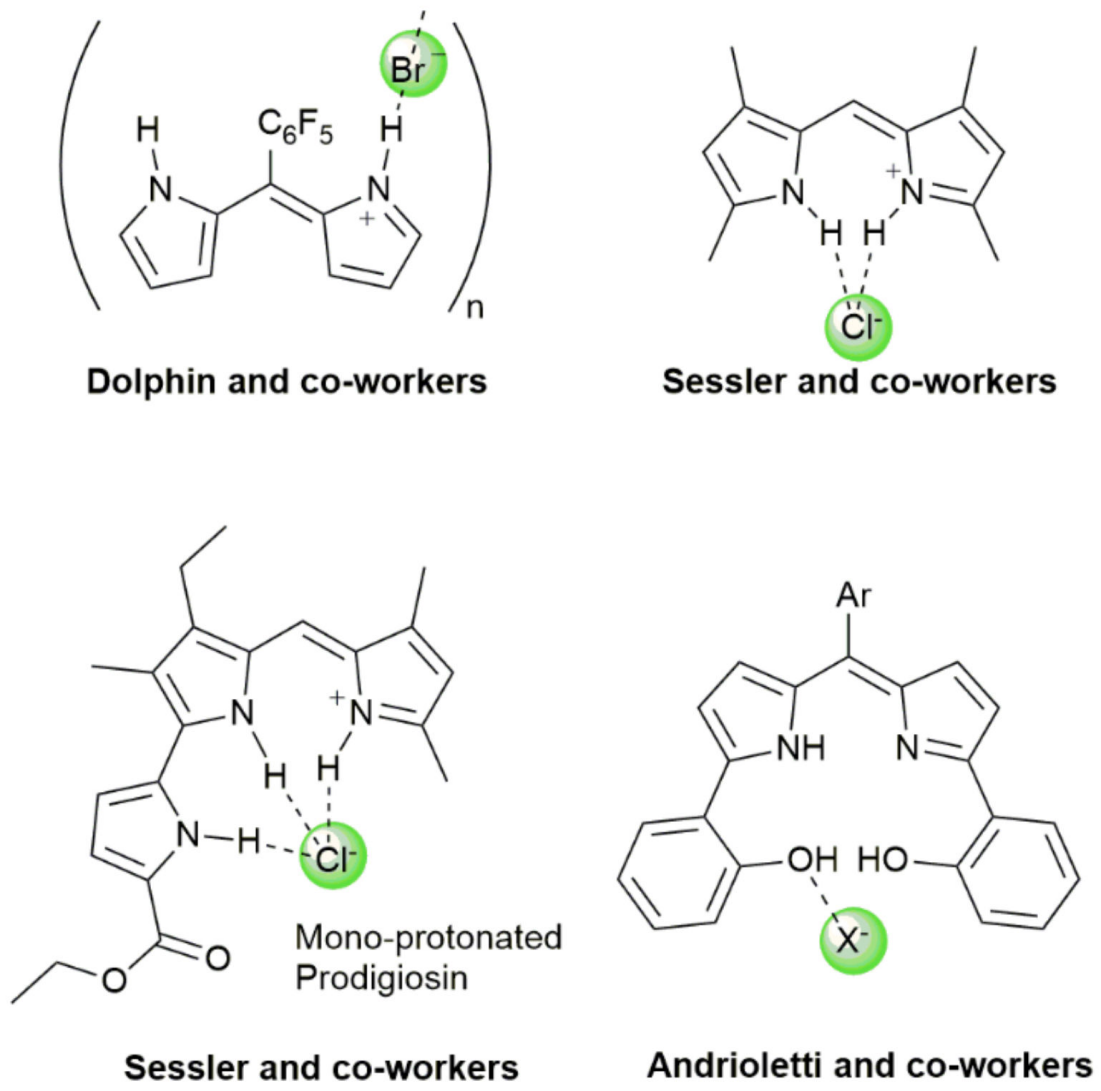
Examples of some dipyrrins involved in anion binding.

In parallel, several groups became interested in the ability of the dipyrromethene motif to coordinate anions (Figure 1) [10]. It is now admitted that only the protonated form of dipyrromethene is active. Hence, Dolphin and co-workers [11] evidenced a hydrogen bond network between the acidic pyrrolic hydrogen and a bromide anion. Sessler, Magda and co-workers demonstrated that prodigiosin displays a low affinity for chloride ions in contrary to its monoprotonated form in which the two pyrrole NHs strongly bind Cl^−^ [12]. Finally, our group recently designed dipyrromethenes substituted at the α-pyrrolic positions by two phenol rings. The recognition of anions such as Cl^−^, Br^−^, HSO_4_^−^, and NO_3_^−^ was possible only through the phenols, without any involvement of the neutral pyrroles [13].

**Figure 2:**
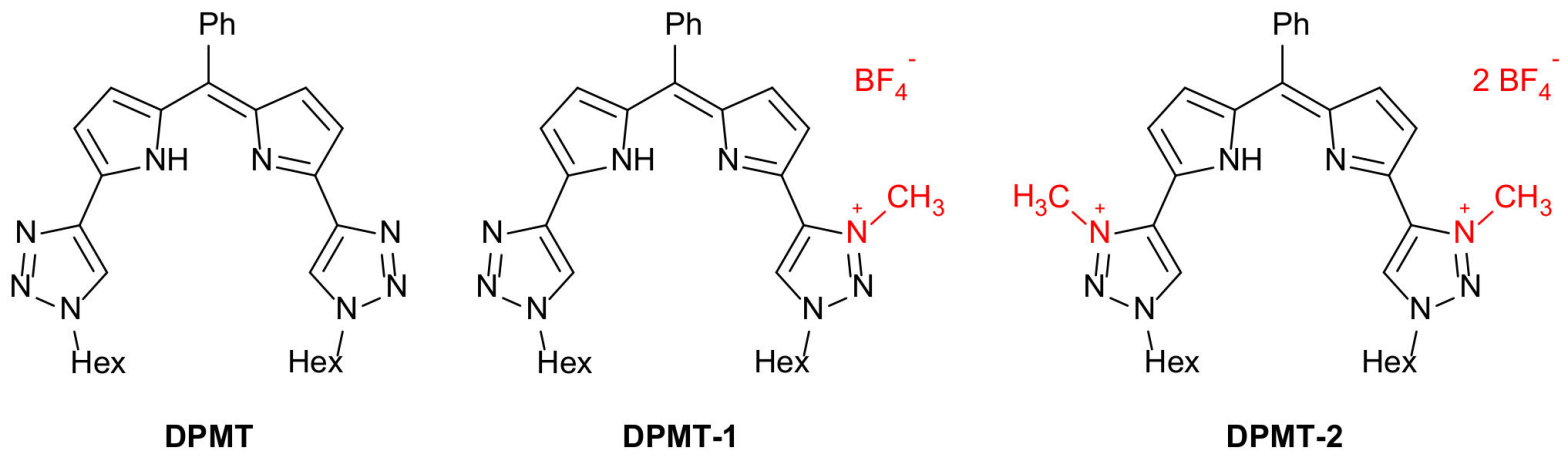
Structures of the targeted bis-triazole dipyrromethene ligands investigated.

During the course of our recent studies on the development of triazole-appended dipyrromethenes (TADs) as powerful fluorescent dyes [14], we developed the synthesis of a dipyrromethene decorated with coordinating triazole heterocycles. Indeed, thanks to the presence of the three nitrogen atoms of the triazole moiety, the C5-H proton becomes acidic and prone to be involved in hydrogen-bonding with anions [15]. In addition, triazoliums are known to be highly efficient in the anion recognition thanks to the even more acidic C5 proton of the quaternarized triazolium ring, which participates in hydrogen bond interactions [16,17]. Consequently, after considering the precedents in the literature, we investigated the bis-triazole dipyrromethene (DPMT) ligand and its derivatives as potential anion receptors (Figure 2).

## 2. Results and Discussion

**Scheme 1:**
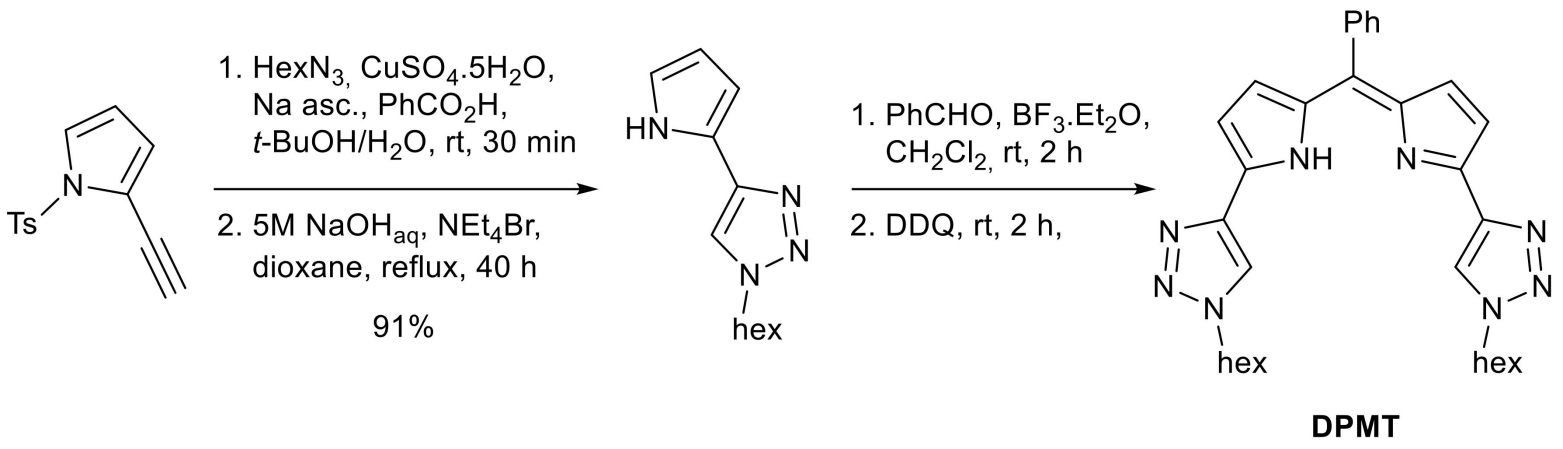
Synthesis of the bis-triazole dipyrromethene structure.

The neutral ligand was synthesized according to the procedure previously described by one of the authors (Scheme 1) [14].

**Scheme 2:**
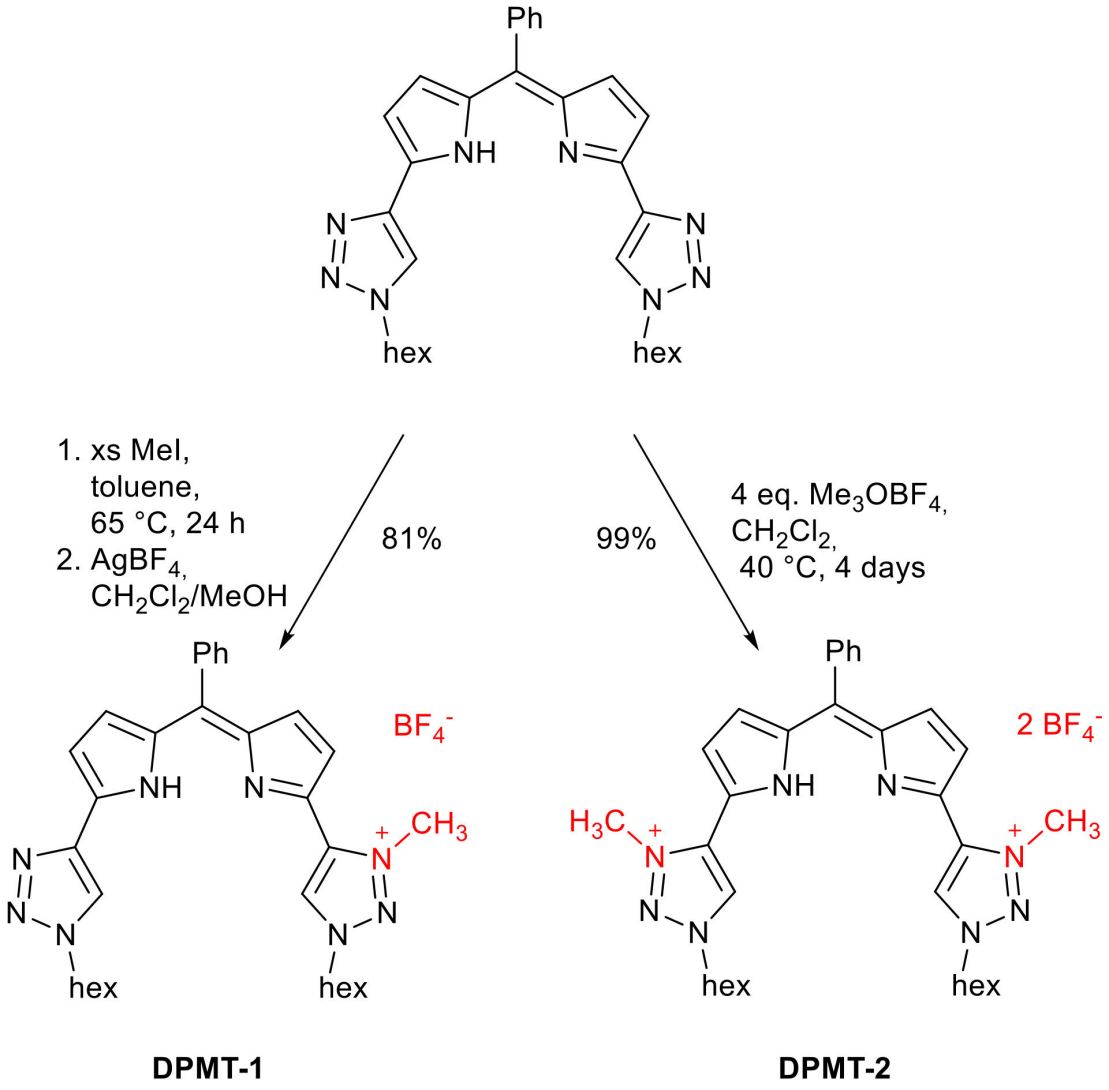
Synthesis of the mono- and bis-triazolium dipyrromethene ligands using either methyl iodide or the Meerwein’s salt.

The mono-alkylated triazolium was prepared by the addition of methyl iodide, while the bis-triazolium was synthesized using the Meerwein’s salt (Scheme 2) [18]. At last, the tetrafluoroborate anion was chosen as the final counter anion, because it is known to generally display low affinity for anion receptors [1,2,3,4].

Several anions in various concentrations were added as tetrabutylammonium salts (TBA^+^X^−^ with X = Cl, F, Br, I, NO_3_, HSO_4_, H_2_PO_4_, CH_3_COO), and the solutions were analyzed by ^1^H NMR titrations in CDCl_3_ (see Supplementary Materials). As expected, the neutral **DPMT** did not display any noticeable affinity for any anions in contrary with **DPMT-1** and **DPMT-2**. Interestingly, the ^1^H-NMR analyses of **DPMT-1** and **DPMT-2** revealed that the C5-H proton of the triazolium ring(s) shifted downfield suggesting its (their) putative involvement in the anion binding. Conversely, no significant change was observed for the C-H of the neutral triazole ring of **DPMT-1**, which indicates that it is not involved in the binding of Cl^−^, as observed with the **DPMT**.

Remarkably, both the **DPMT-1** and **DPMT-2** hosts display 1:1 stoichiometries with the different anions, as indicated by the Job Plots experiments (see the Supplementary Materials). Hence, the two triazoliums do not behave independently in **DPMT-2**. Table 1 gathers the different titration association constants calculated with a series of anions.

**DPMT-1** and **DPMT-2** appeared to be relatively unstable in the presence of acidic (HSO_4_^−^ and H_2_PO_4_^−^) as well as basic (F^−^ and CH_3_COO^−^) anions as the two ligands rapidly degraded in their presence. Possibly, the more basic anions deprotonate the C5-H of the triazolium affording the corresponding free carbenes that degrade in the reaction mixture. Among the other anions studied, Cl^−^ and Br^−^ afforded the highest association constants (Table 1).

**Figure 3:**
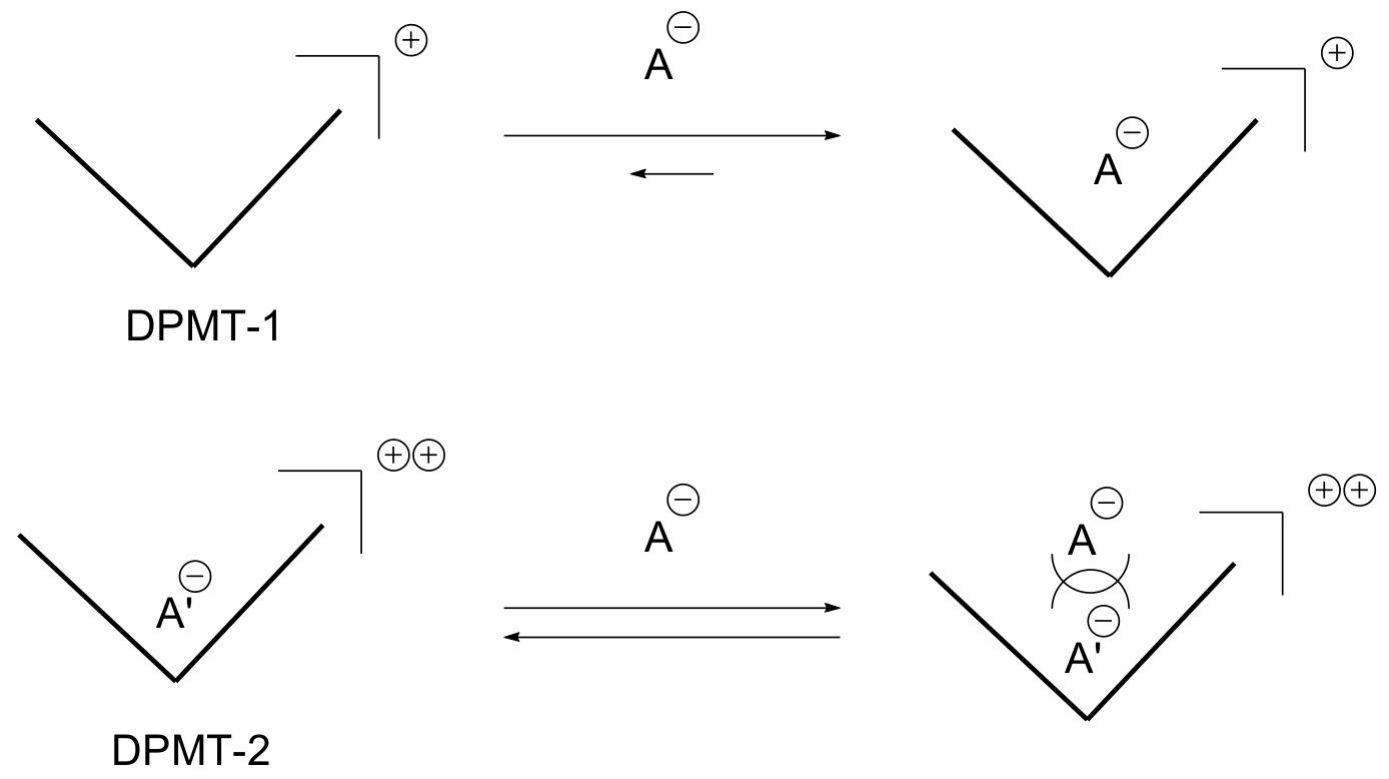
Simplified binding models.

Noticeably, for all the anions, **DPMT-2** displayed slightly lower association constants than those observed with **DPMT-1**. Whilst possibly counter-intuitive, this observation can be explained by the detrimental effect of the firstly bound anion in the cavity of **DPMT-2** (Figure 3), hence disfavoring the coordination of the second guest. In addition, such a hypothesis accounts for the 1:1 stoichiometry. Consequently, in the **DPMT-2** case, the incoming anion has to approach a ligand that already hosts a negatively charged guest.

As already discussed, fluorine-containing anions are usually employed as non-interfering counter anions, but manifold studies revealed the ability of these anions to afford supramolecular assemblies and metal-coordinated complexes [5,6,7,8,9].

To confirm this hypothesis, HOESY ^1^H-^19^F NMR experiments were performed (see the Supplementary Materials). These analyses evidenced heteronuclear through-space connections, between ^1^H and ^19^F in our case. Indeed, two strong NMR cross peaks were observed on the HOESY spectrum of **DPMT-1**. This observation clearly showed the strong interaction between the two CH-triazole/triazolium protons and the nearby fluorine atoms of BF_4_^−^. In the case of **DPMT-2**, two interactions were also observed: one between the CH-triazolium protons and BF_4_^−^ and one between the methyl groups of the methylated triazole rings and BF_4_^−^. These two spectra thus confirmed the strong interaction between BF_4_^−^ and **DPMT-1** and **DPMT-2**.

**Figure 4:**
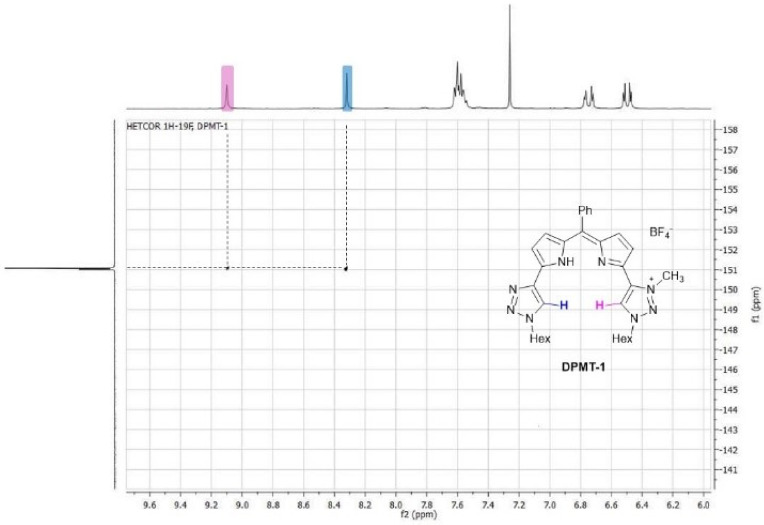
HETCOR ^1^H-^19^F NMR experiment of **DPMT-1** highlighting the “covalent-like” bond between the triazole protons of **DPMT-1** and the counter anion BF_4_^−^.

**Figure 5:**
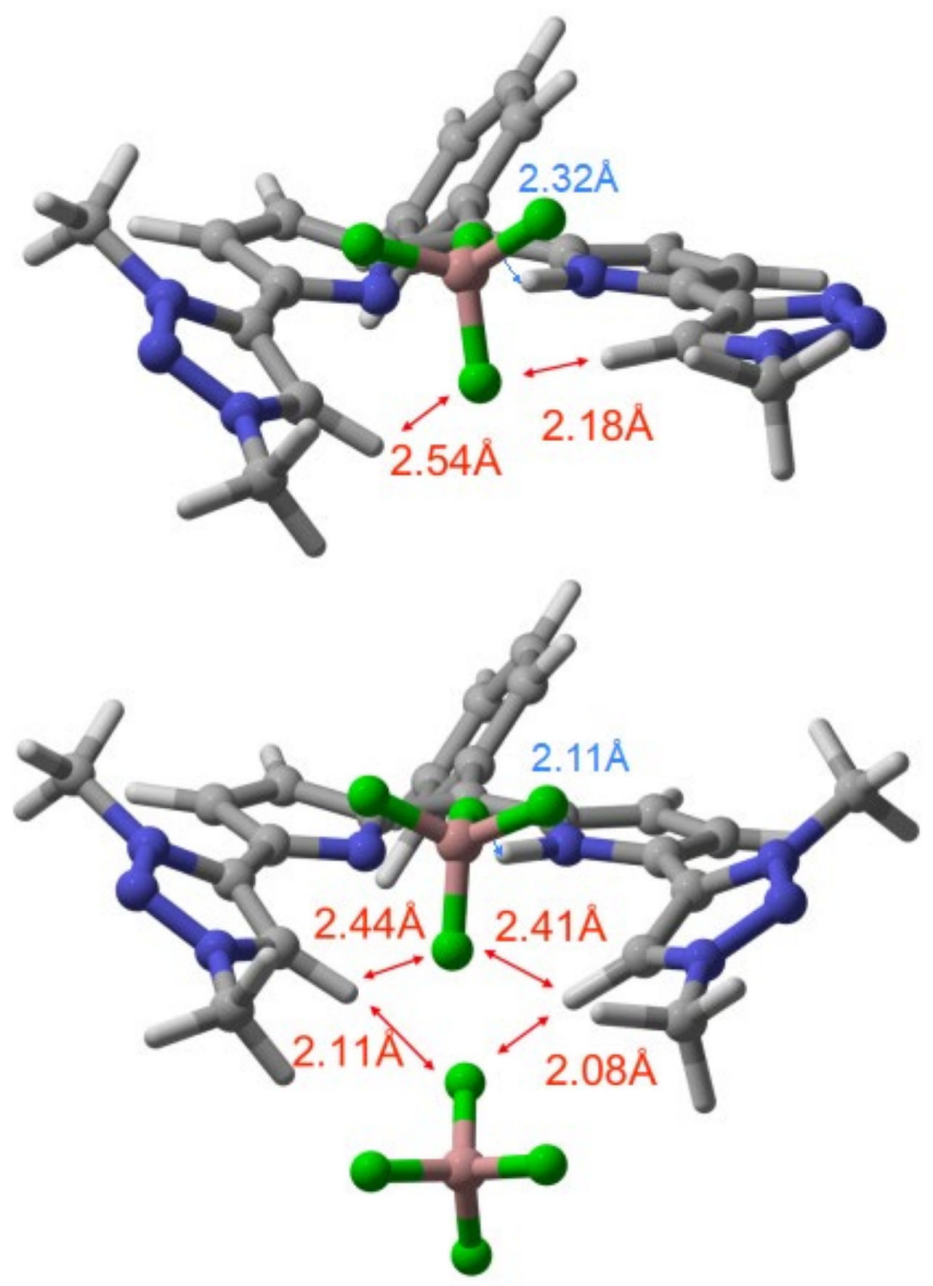
Optimized DFT structures of **DPMT-1**, BF_4_^−^, and **DMPT-2**, (BF_4_^−^)_2_. Distances from the closest F of BF_4_^−^ to the pyrrole NH are shown in blue and to the triazolium CH in red.

Next, HETCOR ^1^H-^19^F NMR experiments were recorded as this 2D experiments allow the detection of heteronuclear connectivity through single-bond couplings. Interestingly, two strong correlation peaks were evidenced on the spectra (Figure 4 and Supplementary Materials). Hence, the non-covalent interaction between the triazole/triazolium protons and the fluorine atoms of BF_4_^−^ is so strong that it can evidenced using HETCOR experiments. This observation was further confirmed by the optimization of the corresponding most stable geometries at the M062X-Def2TZVPP level of theory (Figure 5) using a continuum solvent for the chloroform solvent. **In DPMT-1**, the triazolium ring slightly rotates out off the plane of the dipyrromethene. BF_4_^−^ sits in the resulting cavity, with a distance as low as 2.18 Å to the triazole proton and 2.32 Å from the pyrrole proton. The computed complexation Gibbs Free energy is −66.6 kJ/mol. In **DPMT-2**, the structure of the ligand is even more distorted and forms a pocket where the BF_4_^−^ anion is favorably interacting (ΔG = −118.4 kJ/mol) with the two triazolium protons at a F…H distance of 2.43 Å and 2.29 Å and with the pyrrole proton at a F…H distance of 2.08 Å. To fully neutralize the charge, a second anion may also be interacting with the **DMPT-2** ligand but outside the pocket. It can weakly bind to the pyrrole and the methyl hydrogens with a gain of ΔG = −50.2 kJ/mol. In a more stable configuration (ΔG = −69.6 kJ/mol), the second anion faces the first one from the opposite side of the ligand plane (Figure 5) and interacts with the two triazolium protons with two short F…H distances of 2.11 Å and 2.07 Å.

The association constants calculated in the Table 1 are thus distorted because of the competition between X^−^ and BF_4_^−^ to bind the cation.

**Scheme 3:**
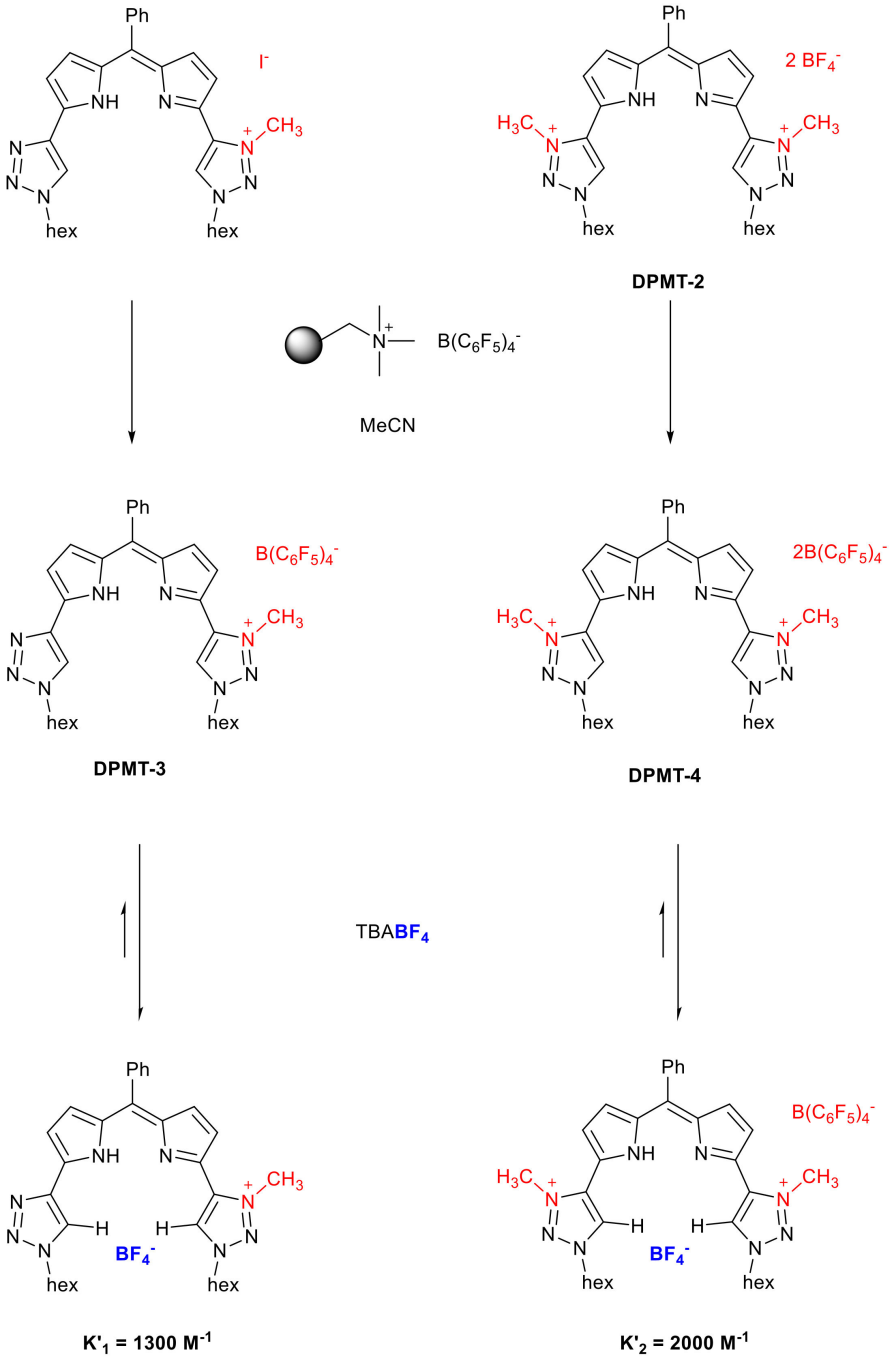
Synthesis of **DPMT-3** and **DPMT-4** containing the non-coordinating counter anion B(C_6_F_5_)^−^ and their association with BF_4_^−^.

The next step was dedicated to the determination of the association constant of BF_4_^−^ to the ligand. Two new ligands **DPMT-3** and **DPMT-4**, displaying B(C_6_F_5_)^−^ as the non-coordinating counter anion, were obtained using an appropriately conditioned ion-exchange resin (Scheme 3). NMR titrations were performed using TBABF_4_ and revealed association constants for BF_4_^−^ of 1300 (**DPMT-3**) and 2000 (**DPMT-4**). These values are relatively important for the binding of an anion such as BF_4_^−^ and justify the lower K observed for **DPMT-2** than **DPMT-1**. Hence, the real association constants K″_1_ and K″_2_, relative to the binding of the anions X^−^ to **DPMT-1** and **DPMT-2**, respectively, were calculated according to the formula: K” = K × K’ (Table 2).

As expected, all the association constants are high (within the 10^5^ M^−1^ range) and meet with reported values concerning charged ligands, but the presence of the two cations within the same molecule is not beneficial, as the association constants are within the same range for both the mono-triazolium host and the bis-triazolium.

## 3. Materials and Methods 

Materials and Methods should be described with sufficient details to allow others to replicate and build on published results. Please note that publication of your manuscript implicates that you must make all materials, data, computer code, and protocols associated with the publication available to readers. Please disclose at the submission stage any restrictions on the availability of materials or information. New methods and protocols should be described in detail while well-established methods can be briefly described and appropriately cited.

## 4. Conclusions

It is a common knowledge that BF_4_^−^ is a weakly coordinating anion. Accordingly, it is often used as a “reference guest” that will be easily displaced by almost any anion. In this communication, we demonstrate that a great care should be taken when assuming that BF_4_^−^ will not interfere. Indeed, in the case of triazole-appended dipyrromethenes, BF_4_^−^ binds so tightly to the TAD scaffold that ^1^H-^19^F NMR cross peaks between the triazole CH protons and the fluorine atoms of BF_4_^−^ are observed. M062X-Def2TZVPP computations in chloroform confirmed that both **DPMT-1** and **DMT-2** adopt a favorable conformation allowing a BF_4_^−^ anion to nicely fit in the TAD cavity in close interaction with the neighboring CH triazole protons. Hence, in the case of the TAD ligands, BF_4_^−^ is not an innocent anion.

## Figures and Tables

**Table 1:** Apparent association constants for the binding of various anions to **DPMT-1** and **DPMT-2** in CDCl_3_.

X^−^ (TBAX)	K_1_ (DPMT-1)	K_2_ (DPMT-2)
F^−^	Degradation	Degradation
Cl^−^	630	164
Br^−^	652	129
I^−^	460	98
NO_3_^−^	444	148
HSO_4_^−^	Degradation	Degradation
H_2_PO_4_^−^	Degradation	Degradation
AcO^−^	Degradation	Degradation

**Table 2:** Corrected association constants for binding of various anions to **DPMT-1** and **DPMT-2** in CDCl_3_.

X^−^ (TBAX)	K_1_ (DPMT-1)	K_2_ (DPMT-2)
Cl^−^	8 × 10^5^	3 × 10^5^
Br^−^	8.5 × 10^5^	2.5 × 10^5^
I^−^	6 × 10^5^	2 × 10^5^
NO_3_^−^	5 × 10^5^	3 × 10^5^

## Supplementary Materials

The following are available online: Syntheses of the compounds, NMR spectra, binding studies including titrations and Job plots experiments as well as computational details.
