# Supplementary material for: Strong Affinity of Triazolium-Appended Dipyrromethenes (TADs) for BF4−
Source: Molecules. 2020 Oct 5;25(19):4555. doi: 10.3390/molecules25194555 (PMC7582478; doi:10.3390/molecules25194555)
Supplement: Supplementary file 1 [file molecules-25-04555-s001.pdf]

Electronic Supplementary Information

for

Strong affinity of Triazolium-Appended-Dipyrromethenes (TADs) for BF_4^-

C. Guerin, Z. Zhang, L. Jean-Gérard, S. Steinmann, C. Michel and B. Andrioletti

Syntheses of the compounds **2**

General Information 2

Syntheses 3

NMR Spectra **7**

Binding studies, general **16**

Titration of the mono-triazolium tetrafluoroborate **17**

Titration of the bis-triazolium bistetrafluoroborate **19**

Job plot experiments **25**

Computational details **27**

Syntheses of the compounds

General Information

^{1}H NMR spectra were recorded on a Bruker AV 400 MHz or 300 MHz spectrometer with reference to residual non-deuterated solvent signal. ^{13}C NMR spectra were recorded at 100 MHz or 75 MHz. ^{19}F NMR spectra were recorded at 376 MHz (relative to $CFCl_3$). The following abbreviations were used to explain multiplicities: s = singlet, d = doublet, t = triplet, q = quartet, m = multiplet, br = broad). The high resolution mass spectra were recorded in a positive and negative ion mode on a hybrid quadrupole time-offlight mass spectrometer (MicroTOFQ-II, Bruker Daltonics, Bremen) with an Electrospray Ionization (ESI) ion source. The gas flow of spray gas is 0.6 bar and the capillary voltage is +/-4,5kV. The solutions are infused at 180µL/h. The mass range of the analysis is 50-1000m/z and the calibration was done with sodium formate. Melting points (mp) were measured on a BUCHI B-540 and are uncorrected.

All reactions were performed under an argon atmosphere in a sealed reaction vial. Reactions were monitored by analytical thin layer chromatography (TLC) using commercial sheets precoated (0.2 mm layer thickness) with silica gel 60F254 (Macherey-Nagel). Product purification by flash column chromatography was performed using Macherey-Nagel Silica Gel (40-63 µm).

Anhydrous solvents were purchased from Sigma Aldrich or Carlo Erba. Other solvents were obtained from commercial sources and used as received.

Preparation of (Z)-1-hexyl-4-(2-((5-(1-hexyl-1H-1,2,3-triazol-4-yl)-1H-pyrrol-2-yl)(phenyl)methylene)-2H-pyrrol-5-yl)-3-methyl-1H-1,2,3-triazol-3-ium iodide

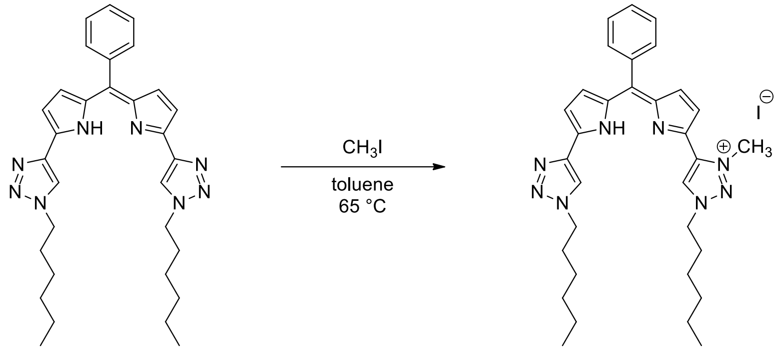

Dipyrromethene (118 mg, 0.225 mmol) was dissolved in toluene (3 mL) and methyl iodide (3 mL) in a sealed-tube. The tube was capped and heated at 65 °C for 24 hours. Next, the mixture was evaporated to dryness to afford a dark solid. The remaining starting material was removed by trituration with toluene (5 mL) followed by filtration on a fritted glass. The trituration/filtration cycle was repeated twice. The filtrate was evaporated to recover the pure starting material which could be recycled. The solid on the fritted glass was collected by dissolution in acetonitrile, then evaporated to dryness to afford the desired mono-triazolium (121 mg, 81% yield) as a dark solid.

^{1}H NMR (400 MHz, DMSO-*d*$_6$): δ = 0.79-0.94 (m, 6H, 2 x CH_3), 1.21-1.43 (m, 12H, 6 x CH_2), 1.83-1.94 (m, 2H, CH_2), 1.94-2.05 (m, 2H, CH_2), 4.47 (t, ^{3}J = 7.0 Hz, 2H, CH_2), 4.67 (t, ^{3}J = 7.2 Hz, 2H, CH_2), 4.83 (s, 3H, C$H_{3,trz}$), 6.72 (d, ^{3}J = 4.0 Hz, 1H, CH_{pyr}), 6.90 (d, ^{3}J = 4.0 Hz, 1H, CH_{pyr}), 6.92 (d, ^{3}J = 4.0 Hz, 1H, CH_{pyr}), 7.18 (d, ^{3}J = 4.0 Hz, 1H, CH_{pyr}), 7.54-7.68 (m, 5H, 5 x CH_{Ph}), 8.83 (s, 1H, CH_{trz}), 9.55 (s, 1H, CH_{trz}), 13.31 (bs, 1H, NH).

^{13}C NMR (100 MHz, DMSO-*d*$_6$): δ = 13.9 (2 x CH$_3$), 21.9 (CH$_2$), 21.9 (CH$_2$), 25.1 (CH$_2$), 25.5 (CH$_2$), 28.4 (CH$_2$), 29.5 (CH$_2$), 30.6 (CH$_2$), 30.6 (CH$_2$), 41.4 (CH$_{3,trz}$), 49.9 (CH$_2$), 53.4 (CH$_2$), 112.9 (CH$_{pyr}$), 122.1 (CH$_{pyr}$), 123.8 (CH$_{trz}$), 128.2 (2 x CH$_{Ph}$), 128.8 (CH$_{pyr}$), 129.5 (CH$_{trz}$), 130.1 (CH$_{Ph}$), 130.8 (2 x CH$_{Ph}$), 133.6 (C_{pyr}), 134.4 (CH$_{pyr}$), 135.8 (C_{Ph}), 136.9 (C_{trz}), 138.4 (C_{pyr}), 138.6 (C_{trz}), 144.8 (C_{meso}), 147.1 (C_{pyr}). 1 carbon missing.

HR-MS (ESI$^+$): [M]$^+$ calcd. for $C_{32}H_{41}N_8$: 537.3449; found: 537.3426.

mp (Buchi): 219 °C.

Preparation of (Z)-1-hexyl-4-(2-((5-(1-hexyl-1H-1,2,3-triazol-4-yl)-1H-pyrrol-2-yl)(phenyl)methylene)-2H-pyrrol-5-yl)-3-methyl-1H-1,2,3-triazol-3-ium tetrafluoroborate (DPMT-1)

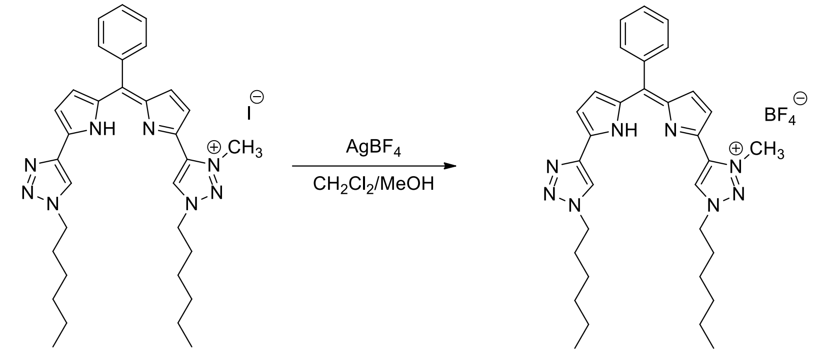

Mono-triazolium I^- (100 mg, 0.15 mmol, 1.0 eq) was dissolved in a mixture of CH_2Cl_2 (5 mL) and MeOH (2 mL). Next, $AgBF_4$ (29 mg, 0.15 mmol, 1.0 eq) was added and the resulting mixture was stirred for 30 min at room temperature. The suspension was concentrated *in vacuo* and dissolved in CH_2Cl_2. After filtration through Celite®, the filtrate was concentrated under vacuum to afford the desired mono-triazolium salt (94 mg, 99% yield) as a red solid.

^{1}H NMR (300 MHz, CDCl$_3$): δ = 0.79-0.95 (m, 6H, 2 x CH_3), 1.22-1.45 (m, 12H, 6 x CH_2), 1.93-2.10 (m, 4H, 2 x CH_2), 4.41 (t, ^3J = 7.4 Hz, 2H, CH_2), 4.63 (t, ^3J = 7.4 Hz, 2H, CH_2), 4.68 (s, 3H, C$H_{3,trz}$), 6.47 (d, ^3J = 4.2 Hz, 1H, CH_{pyr}), 6.51 (d, ^3J = 4.2 Hz, 1H, CH_{pyr}), 6.72 (d, ^3J = 4.3 Hz, 1H, CH_{pyr}), 6.77 (d, ^3J = 4.3 Hz, 1H, CH_{pyr}), 7.51-7.67 (m, 5H, 5 x CH_{Ph}), 8.32 (s, 1H, CH_{trz}), 9.10 (s, 1H, CH_{trz}).

^{13}C NMR (75 MHz, CDCl$_3$): δ = 14.0 (CH$_3$), 14.1 (CH$_3$), 22.5 (CH$_2$), 22.6 (CH$_2$), 26.0 (CH$_2$), 26.3 (CH$_2$), 29.5 (CH$_2$), 30.3 (CH$_2$), 31.0 (CH$_2$), 31.3 (CH$_2$), 42.7 (CH$_{3,trz}$), 50.8 (CH$_2$), 54.3 (CH$_2$), 112.3 (CH$_{pyr}$), 120.8 (CH$_{pyr}$), 123.9 (CH$_{trz}$), 128.3 (2 x CH$_{Ph}$), 128.9 (CH$_{pyr}$), 129.2 (CH$_{trz}$), 130.2(CH$_{Ph}$), 131.0 (2 x CH$_{Ph}$), 133.5 (C_{pyr}), 134.4 (CH$_{pyr}$), 136.2 (C_{Ph}), 137.6 (C_{trz}), 138.7 (C_{pyr}), 138.9 (C_{trz}), 145.7 (C_{meso}), 146.1 (C_{pyr}), 148.0 (C_{pyr}).

^{19}F NMR (376 MHz, CDCl$_3$): δ = -151.0

HR-MS (ESI$^+$): $[M]^+$ calcd. for $C_{32}H_{41}N_8$: 537.3449; found: 537.3451.

mp (Buchi): 222 °C

Preparation of (Z)-1-hexyl-4-(2-((5-(1-hexyl-3-methyl-1H-1,2,3-triazol-3-ium-4-yl)-1H-pyrrol-2-yl)(phenyl)methylene)-2H-pyrrol-5-yl)-3-methyl-1H-1,2,3-triazol-3-ium bis-tetrafluoroborate (DPMT-2)

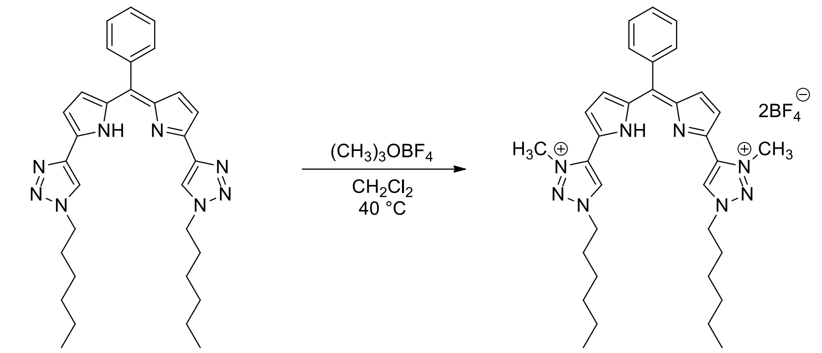

Dipyrromethene (102 mg, 0.195 mmol, 1.0 eq) was dissolved in anhydrous dichloromethane (5 mL) and trimethyloxonium tetrafluoroborate (115 mg, 4 eq) was then added to the solution. The tube was capped and heated at 40 °C for 4 days. The solution was dissolved by addition of acetonitrile (5 mL) and quenched by addition of a solution of saturated aqueous $NaHCO_3$ (500 μL) under vigorous stirring. The mixture was then evaporated to dryness and dissolved in CH_2Cl_2. The solid was filtered off and the filtrate concentrated to afford the expected bis-triazolium salt (136 mg, 99% yield) as a red solid.

^{1}H NMR (400 MHz, DMSO-*d$_6$*): δ = 0.82-0.98 (m, 6H, 2 x CH_3), 1.23-1.47 (m, 12H, 6 x CH_2), 1.88-2.10 (m, 4H, 2 x CH_2), 4.56 (s, 6H, 2 x C$H_{3,trz}$), 4.71 (t, ^3J = 6.9 Hz, 4H, 2 x CH_2), 7.01 (d, ^3J = 4.4 Hz, 2H, 2 x CH_{pyr}), 7.27 (d, ^3J = 4.3 Hz, 2H, 2 x CH_{pyr}), 7.55-7.75 (m, 5H, 5 x CH_{Ph}), 9.46 (s, 2H, 2 x CH_{trz}), 12.53 (bs, 1H, NH).

^{13}C NMR (100 MHz, DMSO-*d$_6$*): δ = 13.9 (2 x CH$_3$), 21.9 (2 x CH$_2$), 25.1 (2 x CH$_2$), 28.5 (2 x CH$_2$), 30.6 (2 x CH$_2$), 40.2 (2 x CH$_{3,trz}$), 53.5 (2 x CH$_2$), 120.7 (2 x CH$_{pyr}$), 128.5 (2 x CH$_{Ph}$), 129.5 (2 x CH$_{trz}$), 130.9 (CH$_{Ph}$), 131.2 (2 x CH$_{Ph}$), 131.6 (2 x CHPh), 135.8 (2 x Ctrz), 136.0 (C_{Ph} or C_{meso}), 138.4 (2 x C_{pyr}), 142.4 (2 x C_{pyr}), 145.7 (C_{Ph} or C_{meso}).

^{19}F NMR (376 MHz, CDCl$_3$): δ = -151.1

HR-MS (ESI$^+$): [M]$^{2+}$ calcd. for $C_{33}H_{44}N_8$: 276.1839; found: 276.1848.

mp (Buchi): 155 °C.

Preparation of the anion $B(C_6F_5)_4^-$ exchange resin

The anion exchange resin (OH^- form) was packed in a column and treated with a solution of MeCN containing 1% of $HClO_4$ (approximatively 5-fold volume). Next, pure MeCN was passed through the column to remove the excess of ClO_4^-. The anionic resin was then washed with a solution of MeCN containing $TBABF_4$, and then washed with a solution of $KB(C_6F_5)_4$.

Preparation of (Z)-1-hexyl-4-(2-((5-(1-hexyl-1H-1,2,3-triazol-4-yl)-1H-pyrrol-2-yl)(phenyl)methylene)-2H-pyrrol-5-yl)-3-methyl-1H-1,2,3-triazol-3-ium tetrakis(pentafluorophenyl)borane (DPMT-3)

The mono-triazolium iodide salt was dissolved in a minimum of MeCN and the solution was eluted through the anion $B(C_6F_5)_4^-$ resin previously prepared. *(This salt proved to be highly unstable, only the ^1H NMR could be recorded)*.

^{1}H NMR (300 MHz, $CDCl_3$): δ = 0.82-0.98 (m, 6H, 2 x CH_3), 1.21-1.47 (m, 12H, 6 x CH_2), 1.86-2.12 (m, 4H, 2 x CH_2), 4.44 (t, ^3J = 7.3 Hz, 2H, CH_2), 4.53 (t, ^3J = 7.3 Hz, 2H, CH_2), 4.98 (s, 3H, $CH_{3,trz}$), 6.54-6.66 (m, 1H, CH_{pyr}), 6.73-6.86 (m, 2H, 2 x CH_{pyr}), 6.93-7.03 (m, 1H, CH_{pyr}), 7.44-7.67 (m, 5H, 5 x CH_{Ph}), 7.89 (br s, 1H, CH_{trz}), 8.40 (br s, 1H, CH_{trz}).

Preparation of (Z)-1-hexyl-4-(2-((5-(1-hexyl-3-methyl-1H-1,2,3-triazol-3-ium-4-yl)-1H-pyrrol-2-yl)(phenyl)methylene)-2H-pyrrol-5-yl)-3-methyl-1H-1,2,3-triazol-3-ium bis tetrakis(pentafluorophenyl)borane (DPMT-4)

The bis-triazolium tetrafluoroborate salt was dissolved in a minimum of MeCN and the solution was eluted through the anion $B(C_6F_5)_4^-$ resin previously prepared. *(This salt proved to be highly unstable, only a ^1H NMR with a poor resolution could be recorded)*.

^{1}H NMR (300 MHz, $CDCl_3$): δ = 0.73-1.00 (m, 6H, 2 x CH_3), 1.15-1.49 (m, 12H, 6 x CH_2), 1.86-2.16 (m, 4H, 2 x CH_2), 4.17-4.91 (m, 10H, 2 x CH_2 + 2 x $CH_{3,trz}$), 6.61-7.17 (m, 4H), 7.37-7.76 (m, 5H, CH_{Ph}), 8.20-8.68 (br s, 1H, CH_{trz}).

NMR Spectra

^{1}H and ^{13}C NMR of the mono-triazolium iodide

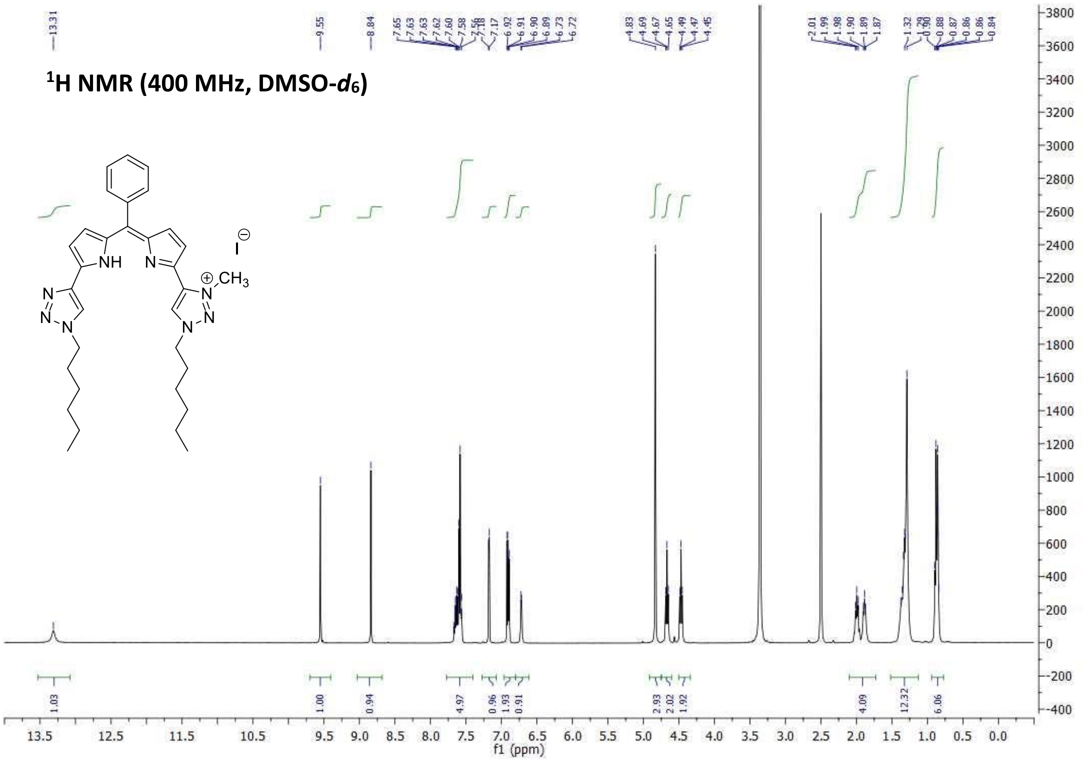

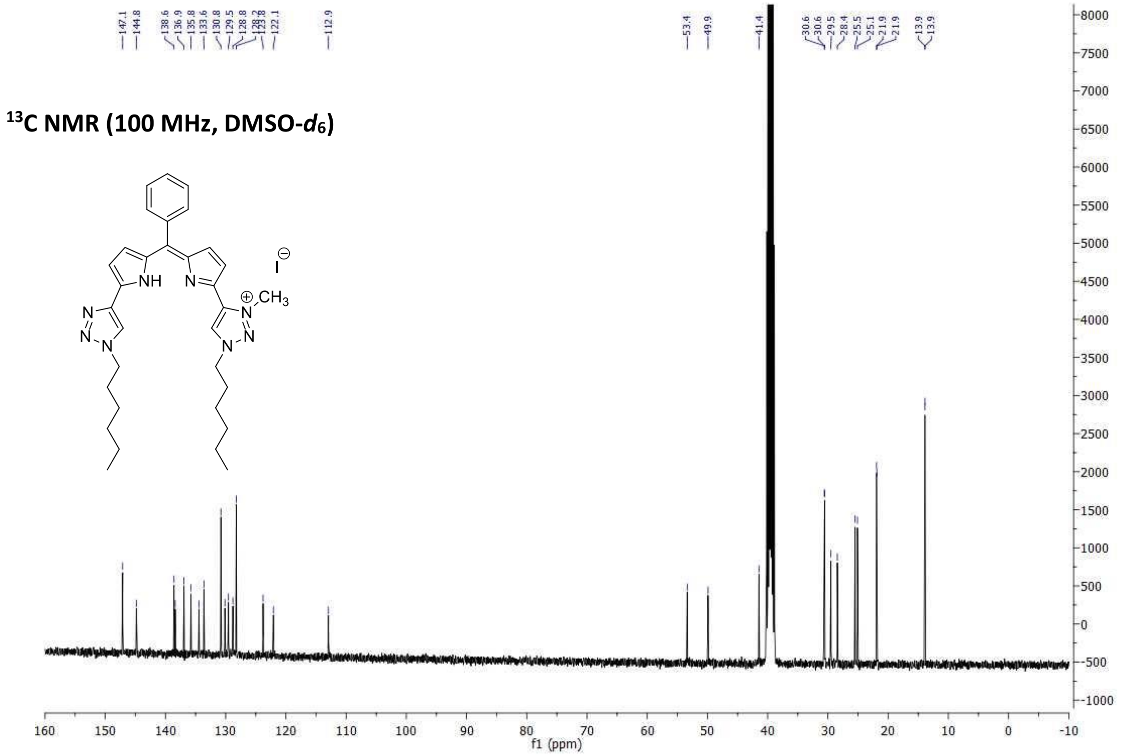

^{1}H, ^{13}C, ^{19}F and HOESY ^{1}H-^{19}F NMR of the mono-triazolium tetrafluoroborate DPMT-1

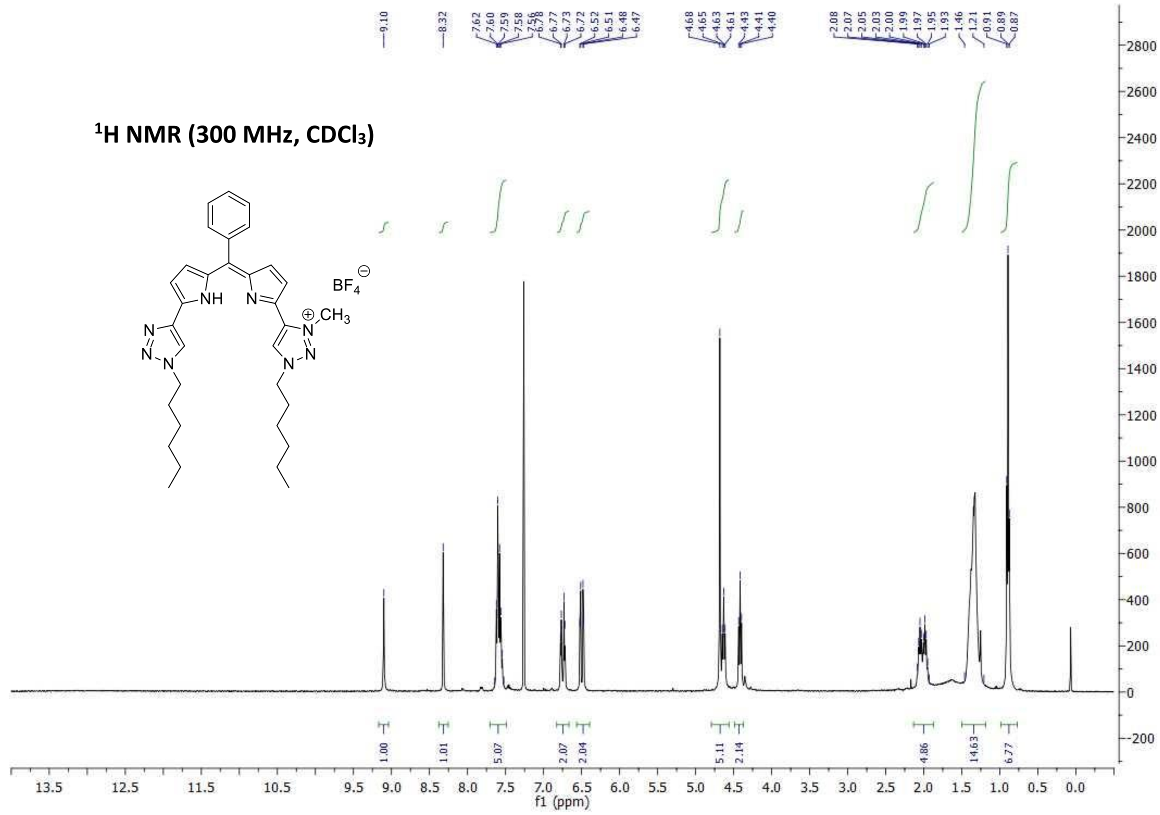

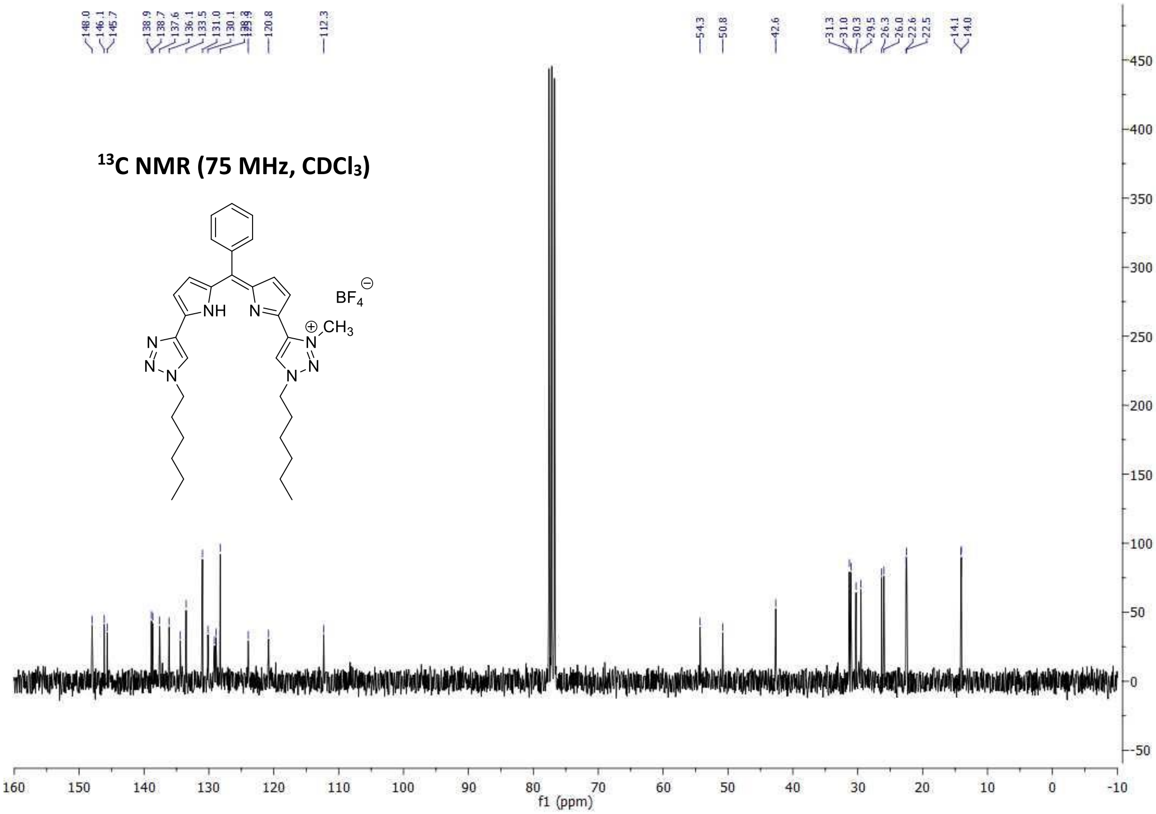

^{19}F NMR (376 MHz, $CDCl_3$)

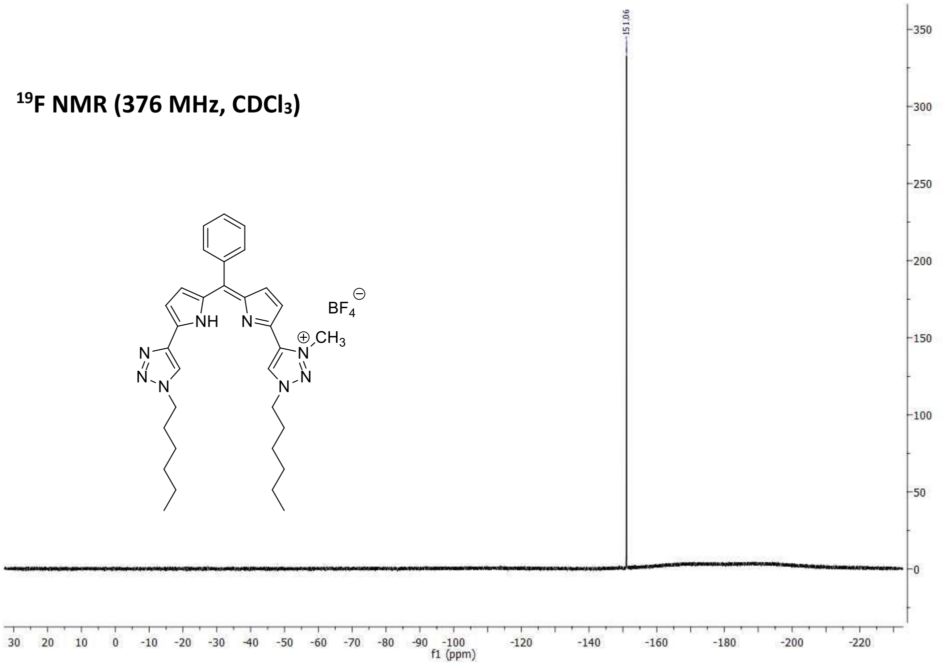

HOESY ^1H-^{19}F NMR ($CDCl_3$)

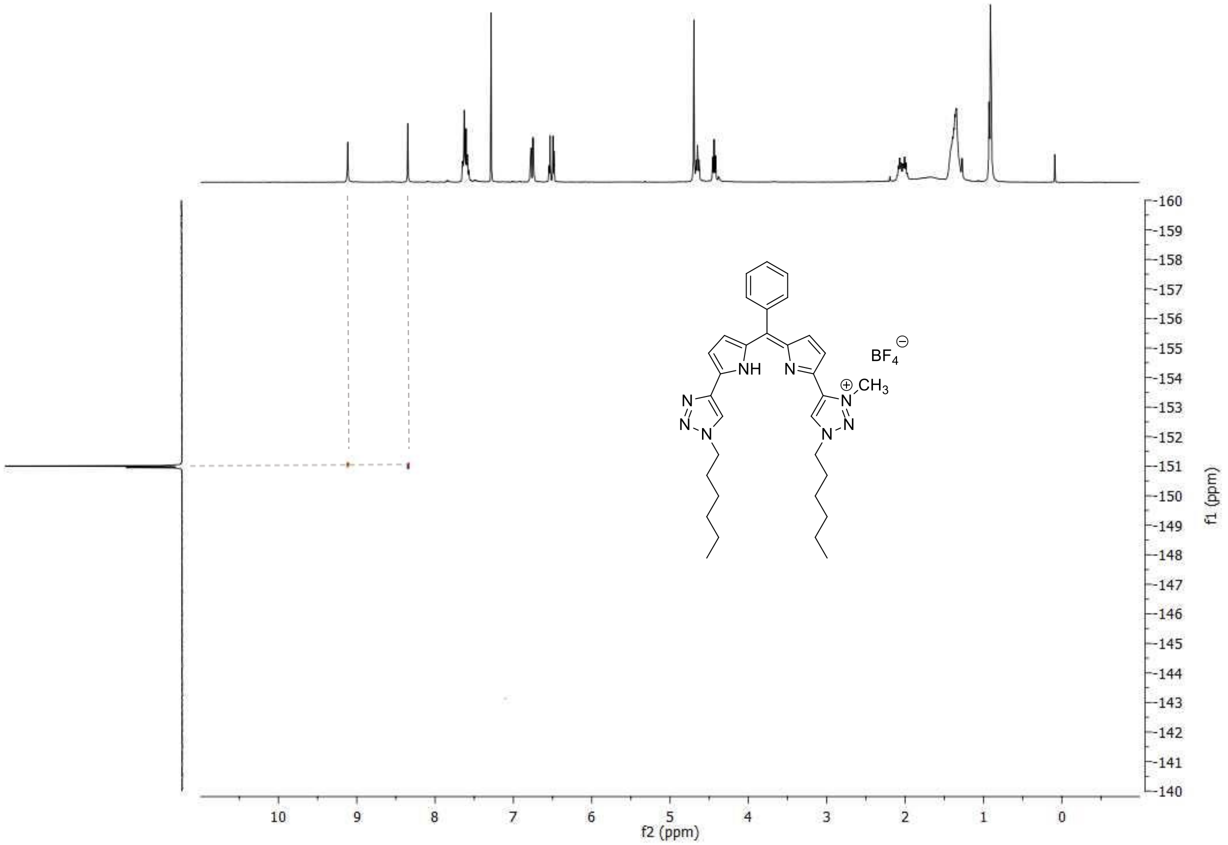

^1H, ^{13}C, ^{19}F, HOESY ^1H-^{19}F NMR and HETCOR ^1H-^{19}F NMR of the bis-triazolium bistetrafluoroborate (DPMT-2)

^1H NMR (400 MHz, DMSO-d_6)

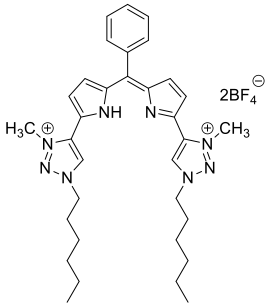

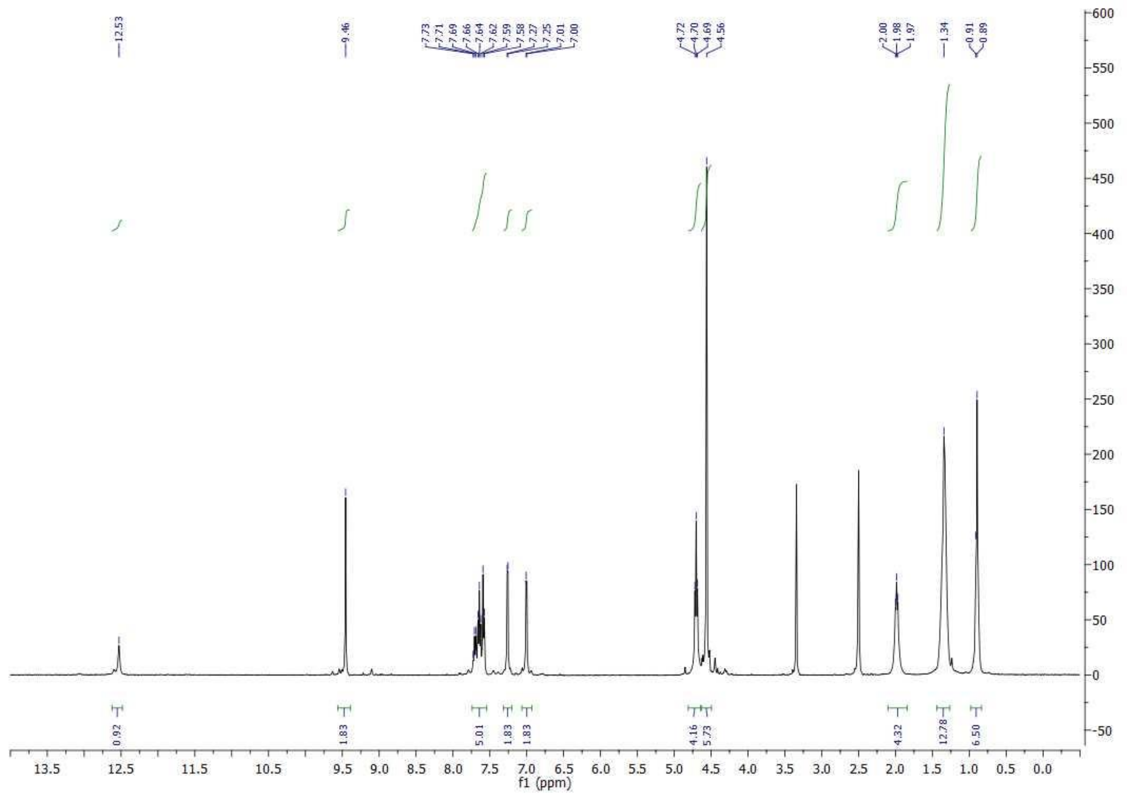
12.53
9.46
7.73
7.71
7.69
7.66
7.64
7.62
7.59
7.58
7.27
7.25
7.01
7.00
4.72
4.70
4.69
4.56
2.00
1.98
1.97
1.34
0.91
0.89
0.92
1.83
5.01
1.83
1.83
4.16
5.73
4.32
12.78
6.50
f1 (ppm)

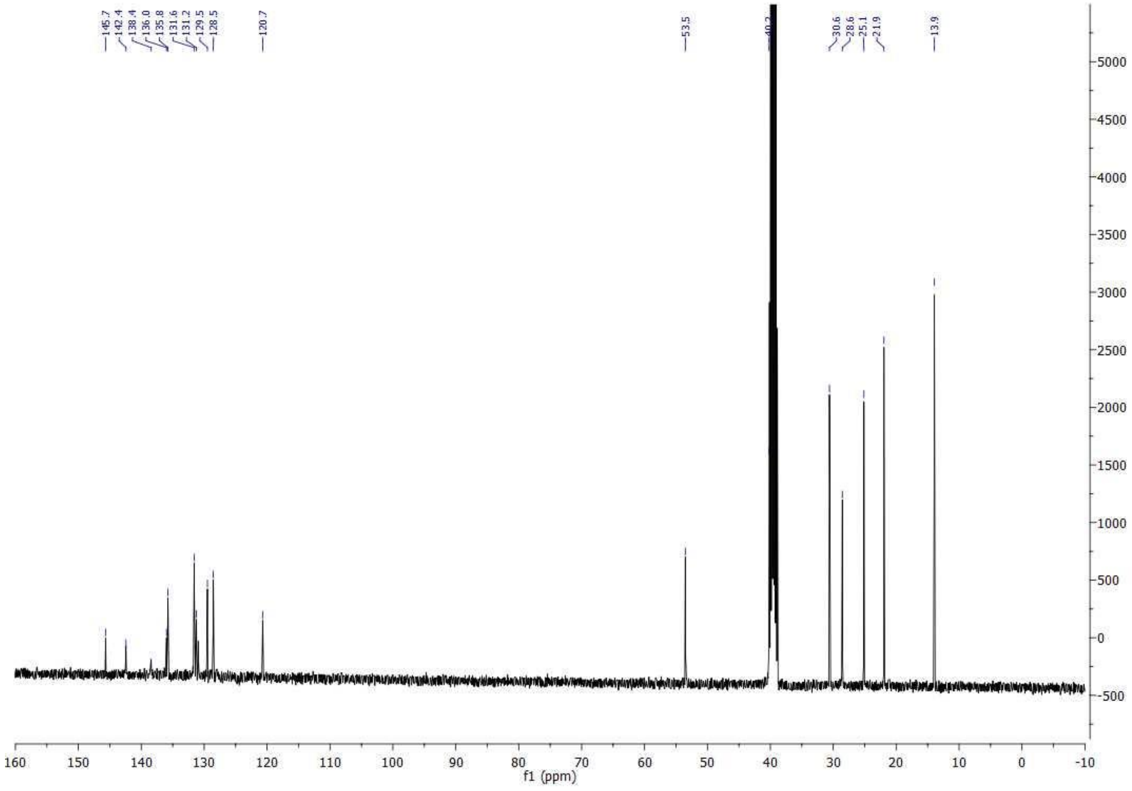
145.7
142.4
138.4
136.0
135.8
131.6
131.2
129.5
128.5
120.7
53.5
30.6
28.6
25.1
21.9
13.9
f1 (ppm)

^{19}F NMR (376 MHz, $CDCl_3$)

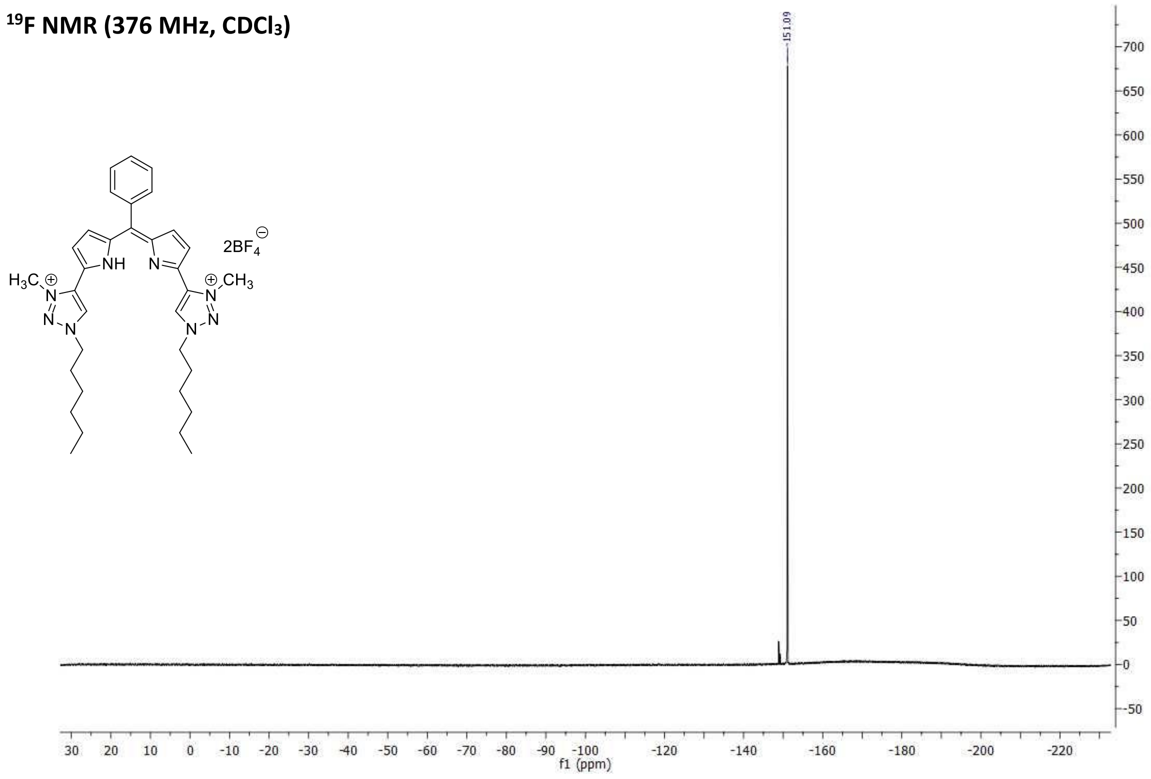

HOESY ^{1}H-^{19}F NMR ($CDCl_3$)

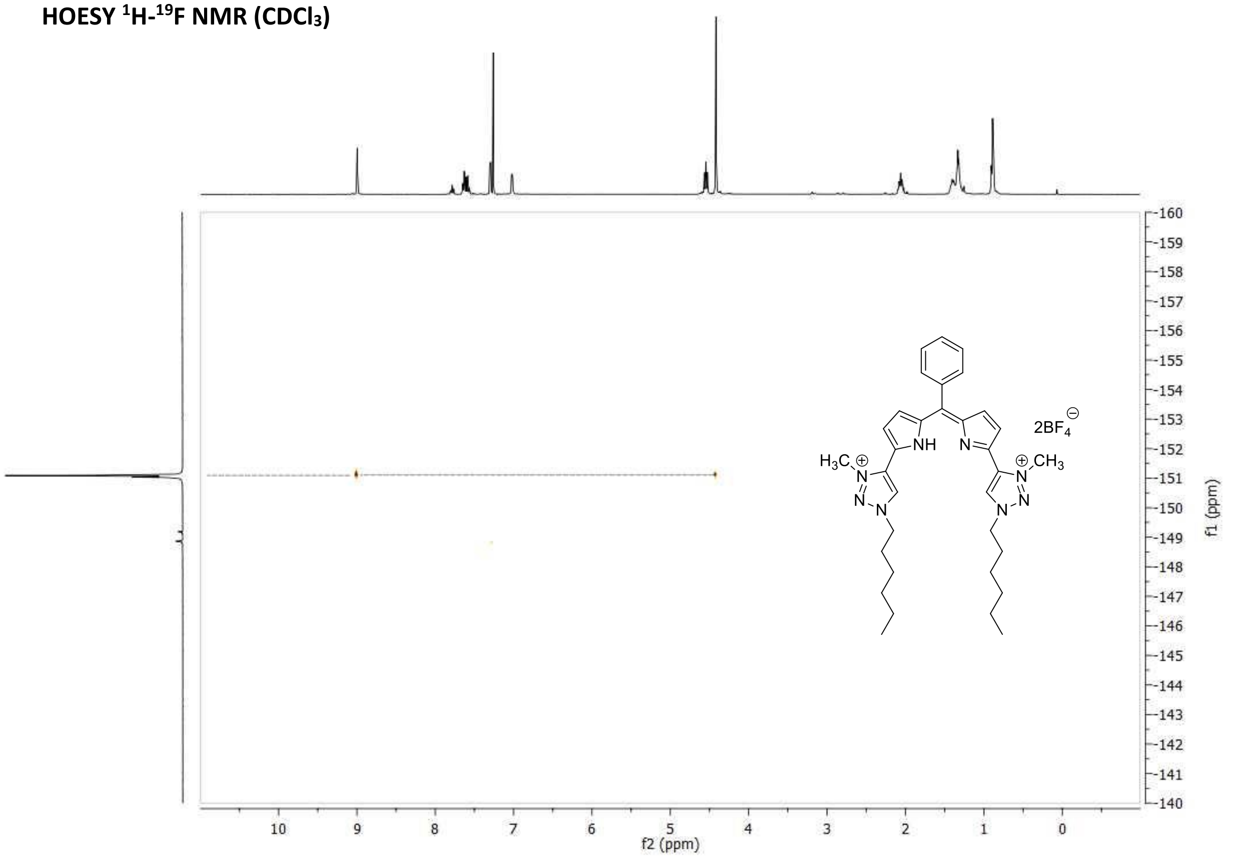

HETCOR ^{1}H-^{19}F NMR ($CDCl_3$)

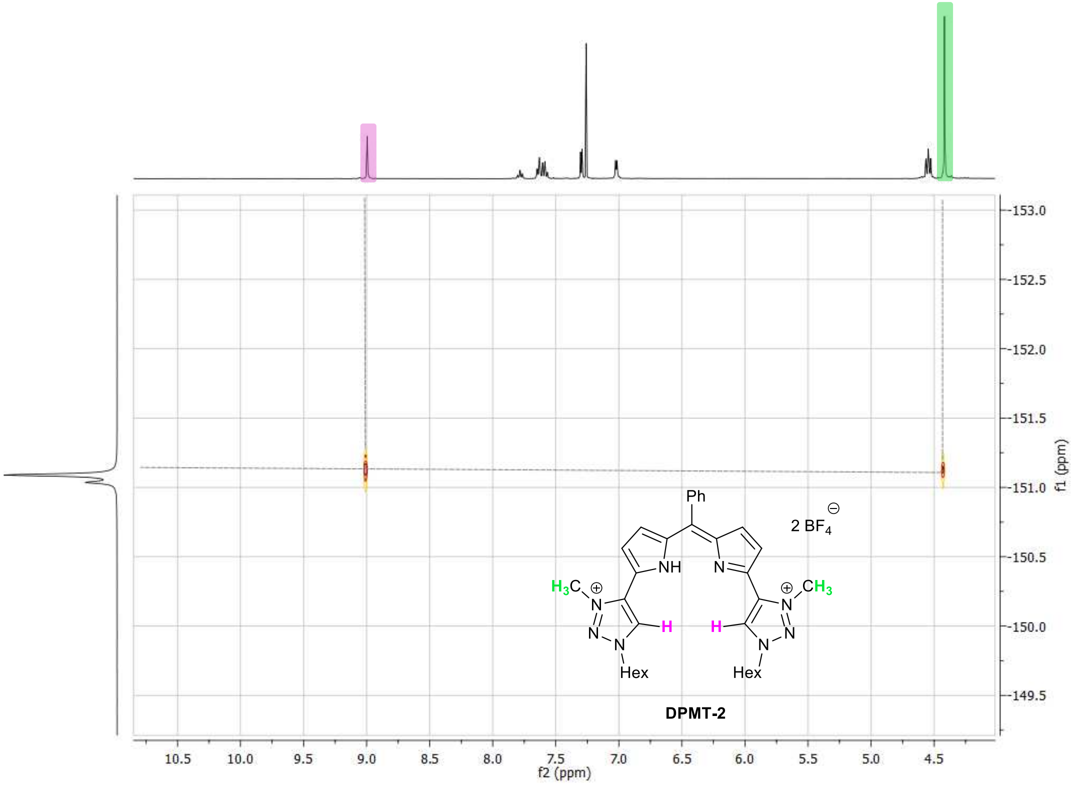

^{1}H NMR of the triazolium tetrakis(pentafluorophenyl)borate DPMT-3

^{1}H NMR (300 MHz, $CDCl_3$)

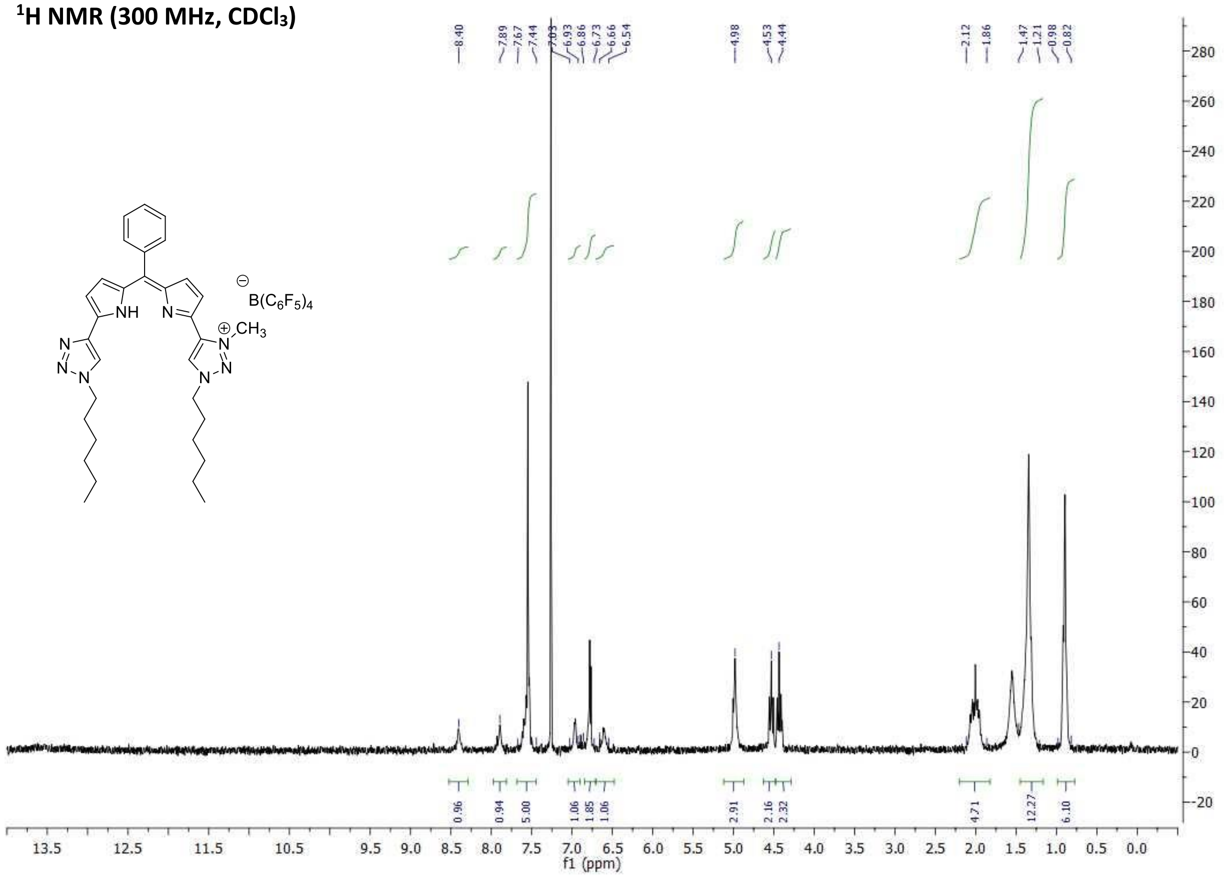

^{1}H NMR of the bis-triazolium bis-tetrakis(pentafluorophenyl)borate DPMT-4

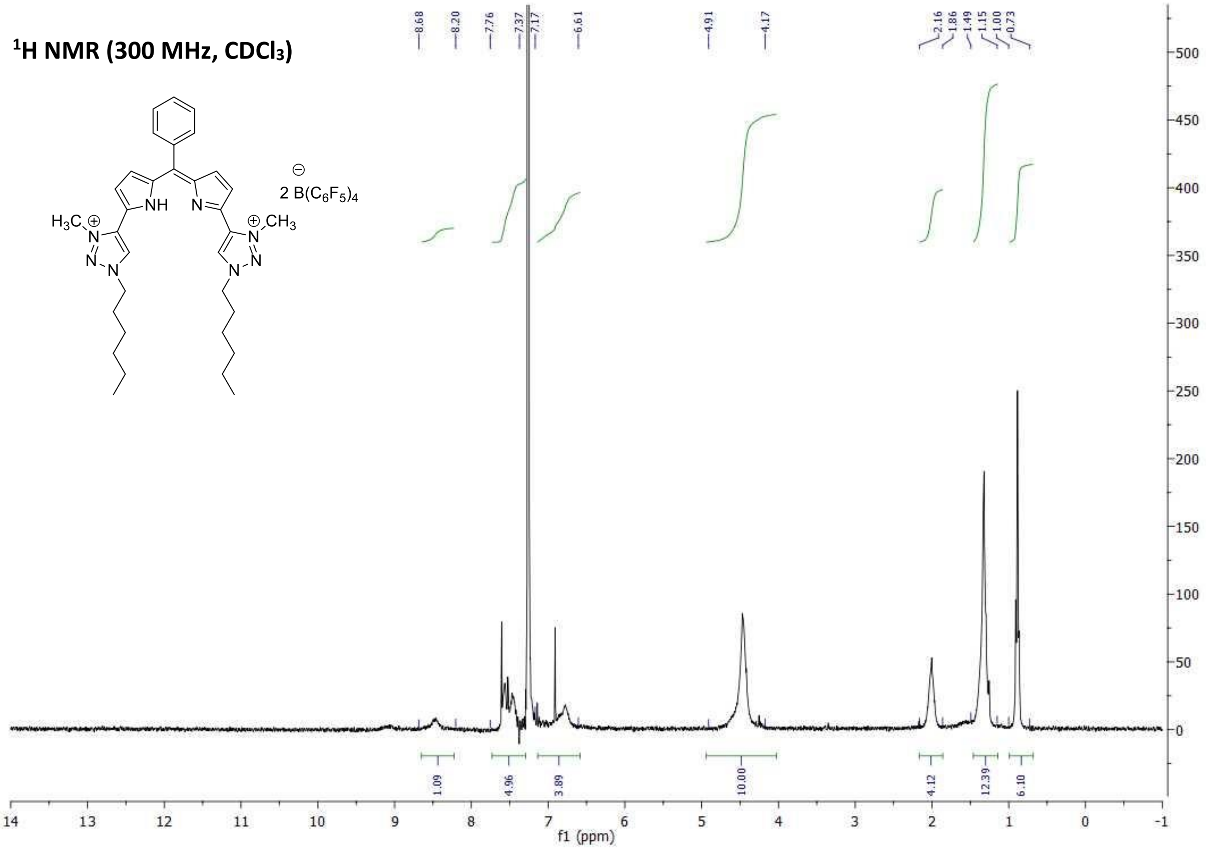

Binding studies, general

^{1}H NMR Titration experiments

The ligand-containing solution (concentration about $5x10^{-3}$ M in $CDCl_3$) was titrated in the NMR tube with the solution of the respective tetrabutylammonium salt (concentration about $125x10^{-3}$ M). Aliquots of the tetrabutylammonium solution were added (from 2,5 µl to 50 µl). The binding constants were calculated from the changes in the chemical shifts of the ligand protons. Nonlinear curve fitting was carried out with Excel. The binding constants K' and the asymptotic change in chemical shift $\Delta\delta_{max}$ were chosen as the free parameters for fitting. The following equation was used to fit experimental data in the case of 1:1 complex:[9,10]

$$\Delta\delta_{th} = \Delta\delta_{max} \cdot \frac{\left(\frac{1}{K} + L_0 + X_0\right) - \sqrt{\left(\frac{1}{K} + L_0 + X_0\right)^2 - 4.L_0.X_0}}{2.L_0}$$

X_0 and L_0 respectively represents the concentration of anion and ligand at t=0. Values of the binding constants obtained with both programs were in agreement.

^{1}H NMR Job plot experiments

NMR tube solutions were prepared by varying the ratio (ligand: anion) with a total constant concentration (20 mM) in $CDCl_3$. 10 points were usually obtained from 10 different solutions with 10 different ratio (ligand: anion) from (4:1) to (1:4).

Binding constants K'

Table 1: Binding constants of the tetrafluoroborate salts in $L.mol^{-1}$

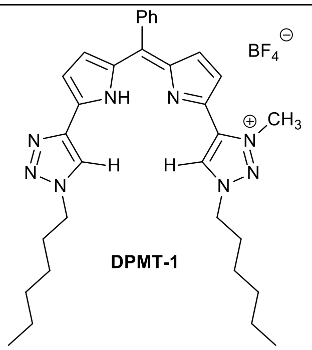

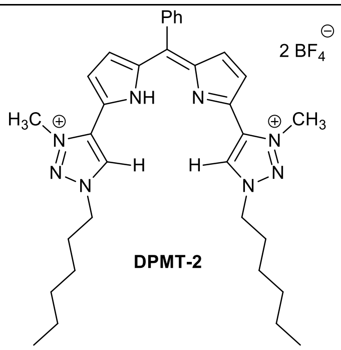

Anion source TBAX	K' (M^{-1})	K' (M^{-1})
F^-	degradation	degradation
Cl^-	630	164
Br^-	652	129
I^-	460	98
NO_3^-	444	148
HSO_4^-	degradation	degradation
$H_2PO_4^-$	degradation	degradation
CH_3COO^-	degradation	degradation

^{1}H NMR Titration curves

1- Titration of the mono-triazolium tetrafluoroborate

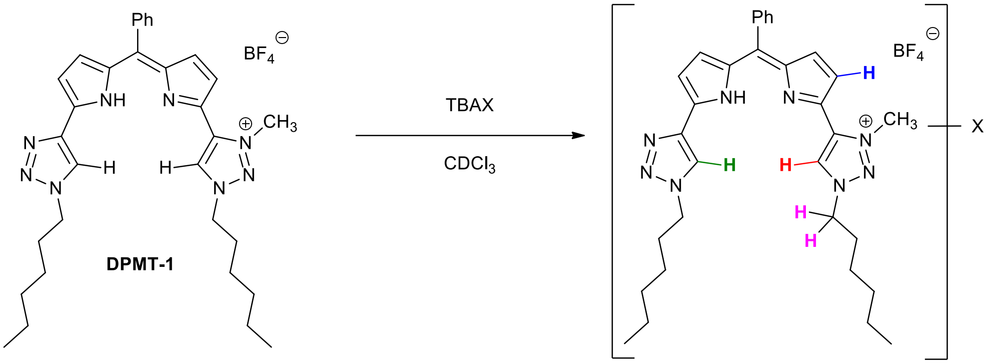

Titration using TBACl

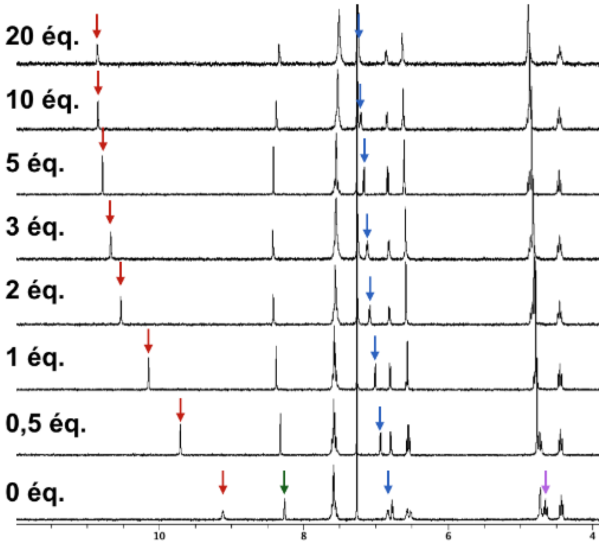

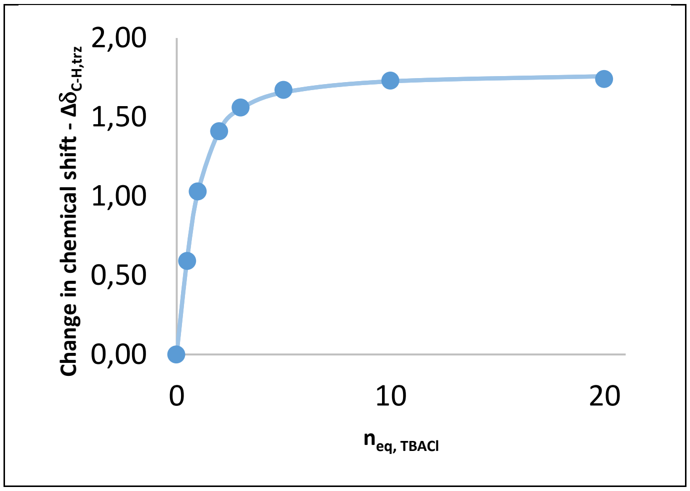

X_0	L_0	Exp. $\Delta\delta_{trz}$	Calc. $\Delta\delta_{trz}$
0	$5{,}00.10^{-3}$	0,00	0,00
$2{,}45.10^{-3}$	$4{,}90.10^{-3}$	0,59	0,61
$4{,}81.10^{-3}$	$4{,}81.10^{-3}$	1,03	1,03
$9{,}27.10^{-3}$	$4{,}63.10^{-3}$	1,41	1,41
$1{,}34.10^{-2}$	$4{,}47.10^{-3}$	1,56	1,55
$2{,}09.10^{-2}$	$4{,}18.10^{-3}$	1,67	1,66
$3{,}59.10^{-2}$	$3{,}59.10^{-3}$	1,73	1,73
$5{,}60.10^{-2}$	$2{,}80.10^{-3}$	1,74	1,76

K = 630 M^{-1}, $\Delta\delta_{max}$ = 1.81 ppm.

Titration using TBABr

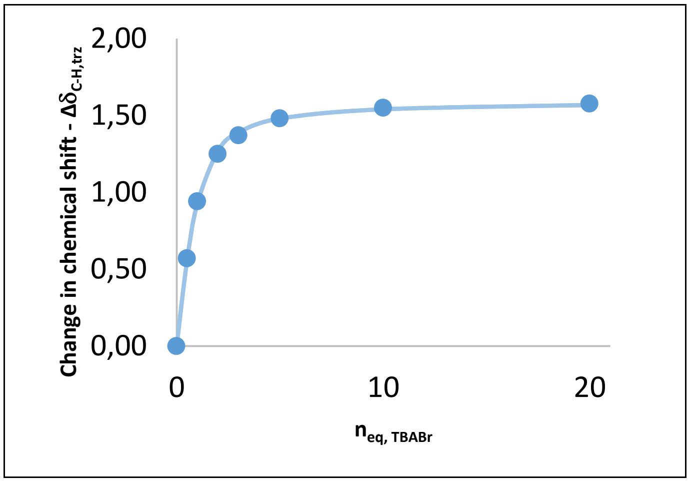

X_0	L_0	Exp. $\Delta\delta_{trz}$	Calc. $\Delta\delta_{trz}$
0	$5,00.10^{-3}$	0,00	0,00
$2,45.10^{-3}$	$4,90.10^{-3}$	0,57	0,55
$4,80.10^{-3}$	$4,80.10^{-3}$	0,94	0,92
$9,23.10^{-3}$	$4,61.10^{-3}$	1,25	1,27
$1,33.10^{-2}$	$4,44.10^{-3}$	1,37	1,39
$2,07.10^{-2}$	$4,14.10^{-3}$	1,48	1,48
$3,53.10^{-2}$	$3,53.10^{-3}$	1,55	1,54
$5,46.10^{-2}$	$2,73.10^{-3}$	1,58	1,57

K = 652 M^{-1}, $\Delta\delta_{max}$ = 1.62 ppm.

Titration using TBAI

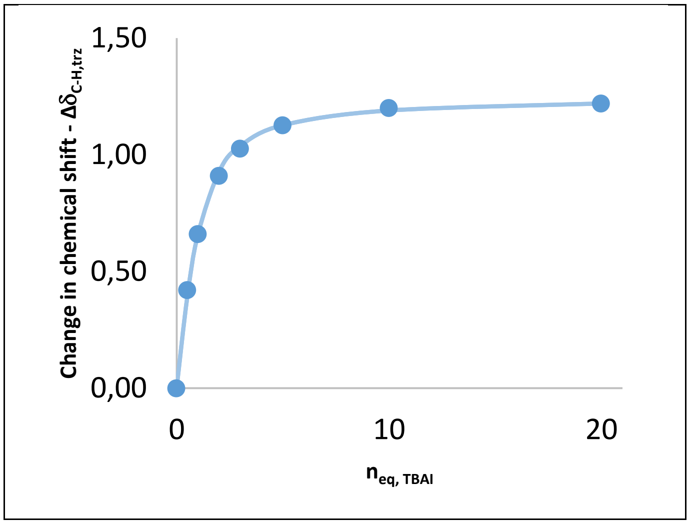

X_0	L_0	Exp. $\Delta\delta_{trz}$	Calc. $\Delta\delta_{trz}$
0	$5,00.10^{-3}$	0,00	0,00
$2,45.10^{-3}$	$4,90.10^{-3}$	0,42	0,39
$4,80.10^{-3}$	$4,80.10^{-3}$	0,66	0,66
$9,23.10^{-3}$	$4,61.10^{-3}$	0,91	0,93
$1,33.10^{-2}$	$4,44.10^{-3}$	1,03	1,04
$2,07.10^{-2}$	$4,14.10^{-3}$	1,13	1,13
$3,53.10^{-2}$	$3,53.10^{-3}$	1,20	1,19
$5,46.10^{-2}$	$2,73.10^{-3}$	1,22	1,22

K = 460 M^{-1}, $\Delta\delta_{max}$ = 1.27 ppm.

Titration using $TBANO_3$

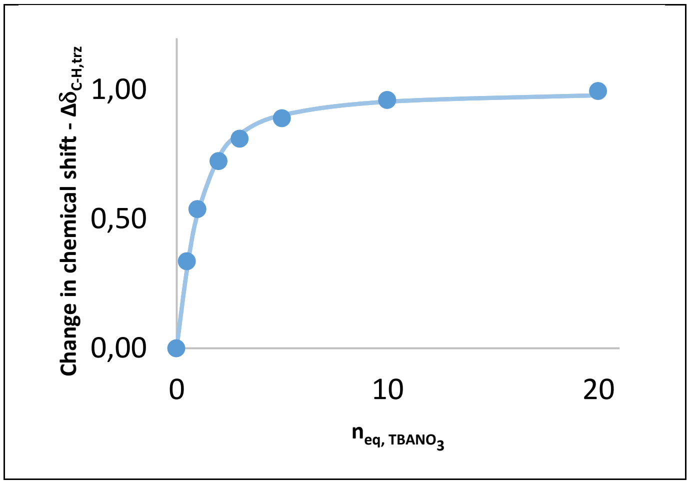

X_0	L_0	Exp. $\Delta\delta_{trz}$	Calc. $\Delta\delta_{trz}$
0	$5,00.10^{-3}$	0,00	0,00
$2,45.10^{-3}$	$4,91.10^{-3}$	0,34	0,31
$4,82.10^{-3}$	$4,82.10^{-3}$	0,54	0,52
$9,29.10^{-3}$	$4,65.10^{-3}$	0,72	0,74
$1,35.10^{-2}$	$4,49.10^{-3}$	0,81	0,83
$2,10.10^{-2}$	$4,20.10^{-3}$	0,89	0,90
$3,63.10^{-2}$	$3,63.10^{-3}$	0,96	0,95
$5,69.10^{-2}$	$2,85.10^{-3}$	1,00	0,98

$K = 444\ M^{-1}$, $\Delta\delta_{max} = 1.02$ ppm.

2- Titration of the bis-triazolium bistetrafluoroborate

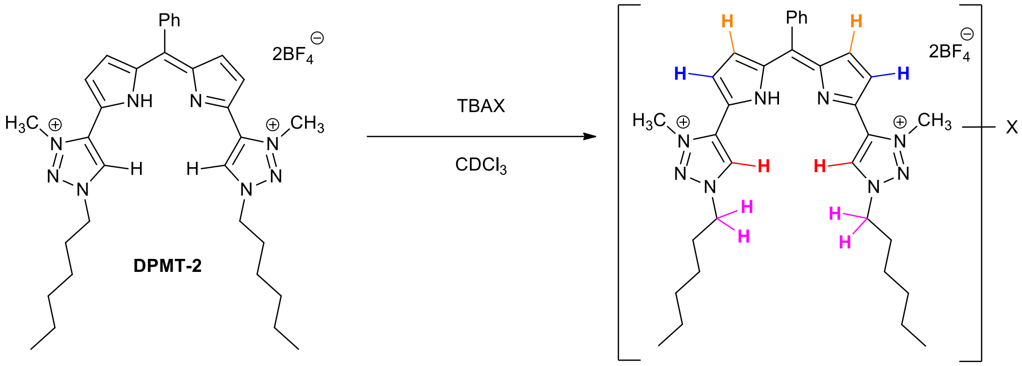

Titration using TBACl

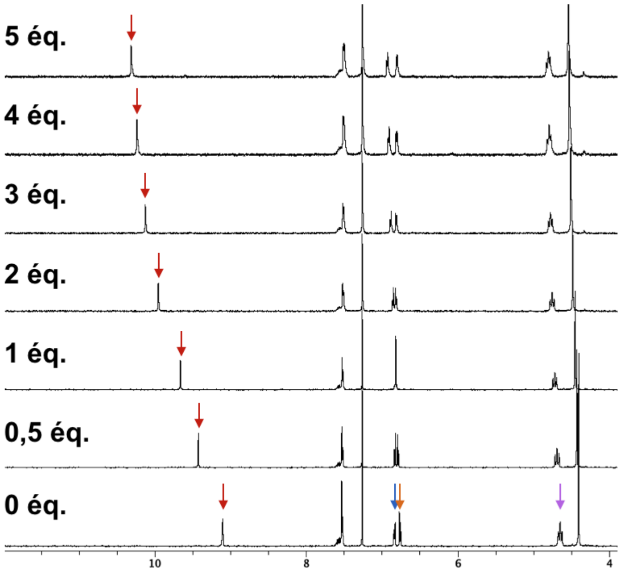

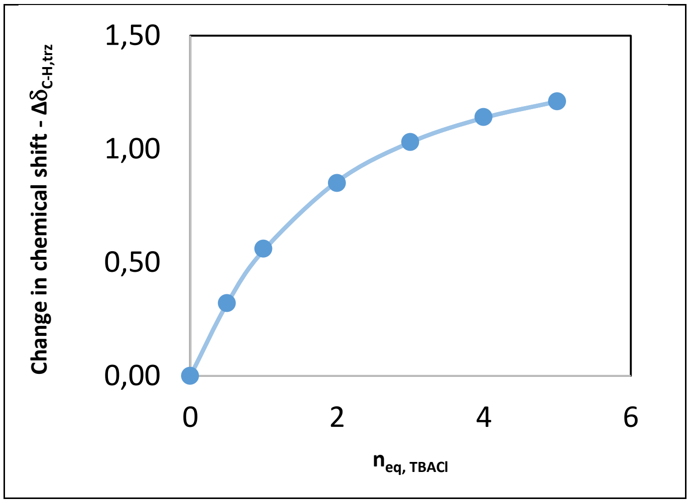

X_0	L_0	Exp. $\Delta\delta_{trz}$	Calc. $\Delta\delta_{trz}$
0	$4{,}98.10^{-3}$	0,00	0,00
$2{,}45.10^{-3}$	$4{,}89.10^{-3}$	0,32	0,31
$4{,}80.10^{-3}$	$4{,}80.10^{-3}$	0,56	0,55
$9{,}26.10^{-3}$	$4{,}63.10^{-3}$	0,85	0,86
$1{,}34.10^{-2}$	$4{,}47.10^{-3}$	1,03	1,03
$1{,}73.10^{-2}$	$4{,}33.10^{-3}$	1,14	1,14
$2{,}09.10^{-2}$	$4{,}19.10^{-3}$	1,21	1,21

K = 164 M^{-1}, $\Delta\delta_{max}$ = 1.62 ppm.

Titration using TBABr

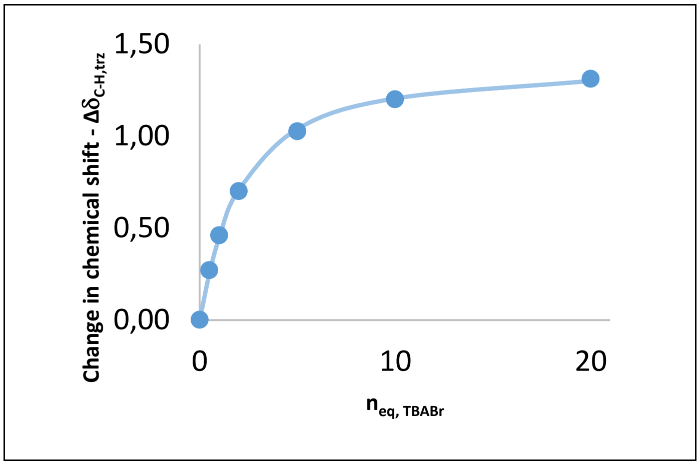

X_0	L_0	Exp. $\Delta\delta_{trz}$	Calc. $\Delta\delta_{trz}$
0	$4{,}98.10^{-3}$	0,00	0,00
$2{,}45.10^{-3}$	$4{,}90.10^{-3}$	0,27	0,25
$4{,}82.10^{-3}$	$4{,}82.10^{-3}$	0,46	0,45
$9{,}32.10^{-3}$	$4{,}66.10^{-3}$	0,70	0,71
$2{,}12.10^{-2}$	$4{,}24.10^{-3}$	1,03	1,04
$3{,}70.10^{-2}$	$3{,}70.10^{-3}$	1,20	1,20
$5{,}88.10^{-2}$	$2{,}94.10^{-3}$	1,31	1,30

K = 129 M^{-1}, $\Delta\delta_{max}$ = 1.48 ppm.

Titration using TBAI

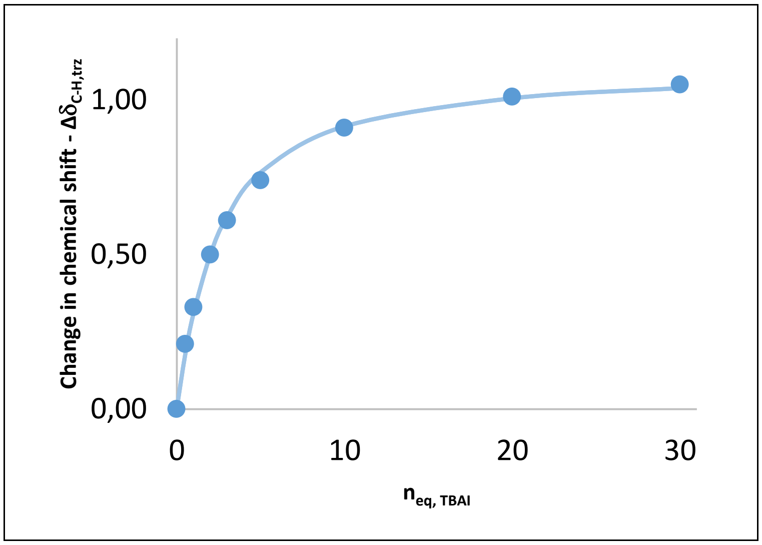

X_0	L_0	Exp. $\Delta\delta_{trz}$	Calc. $\Delta\delta_{trz}$
0	$4{,}99.10^{-3}$	0,00	0,00
$2{,}45.10^{-3}$	$4{,}90.10^{-3}$	0,21	0,17
$4{,}82.10^{-3}$	$4{,}82.10^{-3}$	0,33	0,31
$9{,}31.10^{-3}$	$4{,}65.10^{-3}$	0,50	0,50
$1{,}35.10^{-2}$	$4{,}50.10^{-3}$	0,61	0,62
$2{,}11.10^{-2}$	$4{,}23.10^{-3}$	0,74	0,77
$3{,}66.10^{-2}$	$3{,}66.10^{-3}$	0,91	0,91
$5{,}79.10^{-2}$	$2{,}89.10^{-3}$	1,01	1,01
$7{,}17.10^{-2}$	$2{,}39.10^{-3}$	1,05	1,04

K = 98 M^{-1}, $\Delta\delta_{max}$ = 1.19 ppm.

Titration using $TBANO_3$

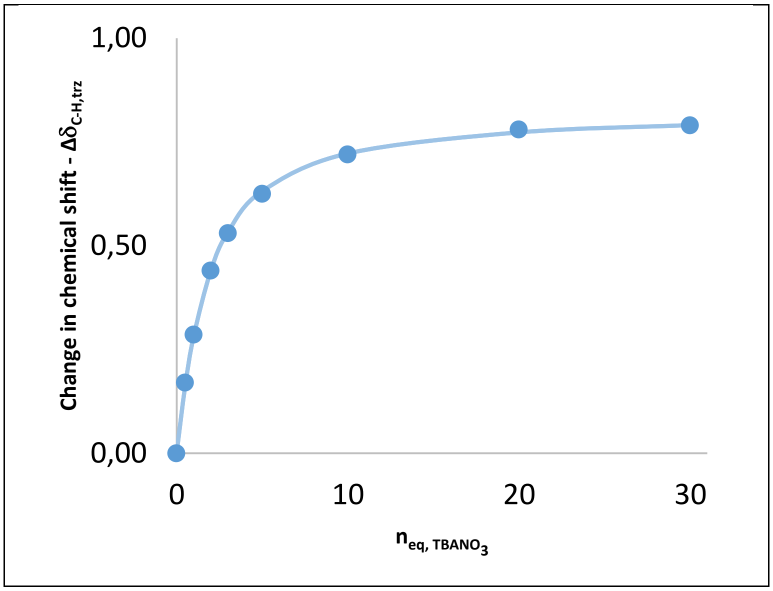

X_0	L_0	Exp. $\Delta\delta_{trz}$	Calc. $\Delta\delta_{trz}$
0	$4{,}99.10^{-3}$	0,00	0,00
$2{,}45.10^{-3}$	$4{,}90.10^{-3}$	0,17	0,16
$4{,}81.10^{-3}$	$4{,}81.10^{-3}$	0,29	0,28
$9{,}31.10^{-3}$	$4{,}65.10^{-3}$	0,44	0,44
$1{,}35.10^{-2}$	$4{,}50.10^{-3}$	0,53	0,53
$2{,}11.10^{-2}$	$4{,}23.10^{-3}$	0,63	0,63
$3{,}67.10^{-2}$	$3{,}67.10^{-3}$	0,72	0,72
$5{,}80.10^{-2}$	$2{,}90.10^{-3}$	0,78	0,77
$7{,}19.10^{-2}$	$2{,}40.10^{-3}$	0,79	0,79

K = 148 M^{-1}, $\Delta\delta_{max}$ = 0.87 ppm.

*Table 2: Binding constants of the **tetrakis(pentafluorophenyl)borane** salts in L.mol^{-1}*

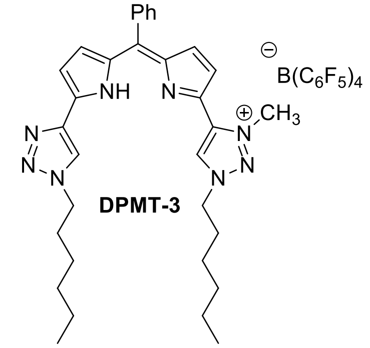

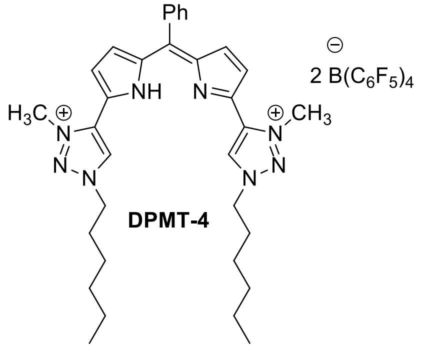

**Anion source TBAX**	**DPMT-3**	**DPMT-4**
	**K' (M^{-1})**	**K' (M^{-1})**
BF_4^-	1300	2000

^{1}H NMR Titration curves

Titration of the mono-triazolium BPh_4 using $TBABF_4$

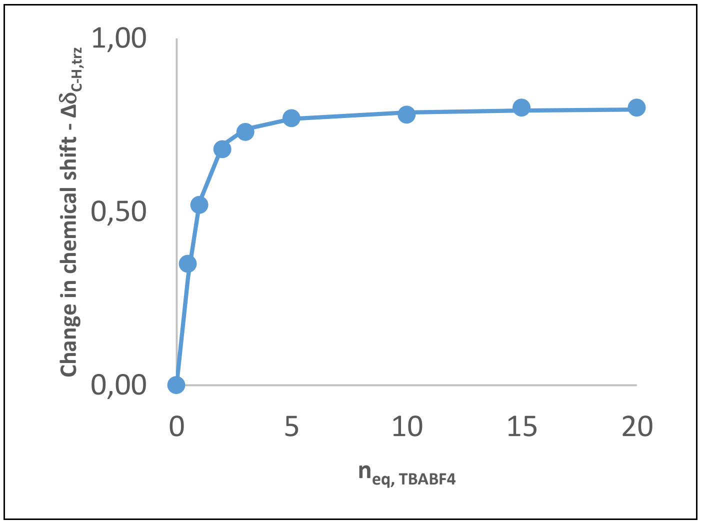

X_0	L_0	Exp. $\Delta\delta_{trz}$	Calc. $\Delta\delta_{trz}$
0	4,24.10^{-3}	0,00	0,00
2,08.10^{-3}	4,17.10^{-3}	0,35	0,31
4,09.10^{-3}	4,09.10^{-3}	0,52	0,53
7,89.10^{-3}	3,95.10^{-3}	0,68	0,69
1,14.10^{-2}	3,81.10^{-3}	0,73	0,74
1,78.10^{-2}	3,57.10^{-3}	0,77	0,77
3,08.10^{-2}	3,08.10^{-3}	0,78	0,79
4,06.10^{-2}	2,71.10^{-3}	0,80	0,79
4,83.10^{-2}	2,42.10^{-3}	0,80	0,80

K = 1300 M^{-1}, $\Delta\delta_{max}$ = 0.81 ppm.

Titration of the bis-triazolium bis-BPh_4 using $TBABF_4$

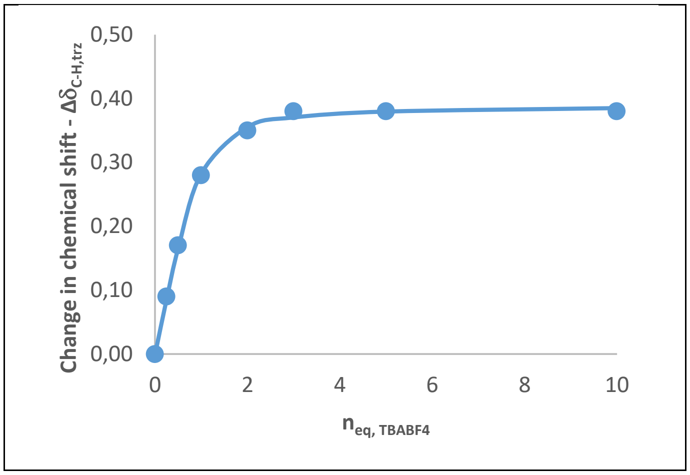

X_0	L_0	Exp. $\Delta\delta_{trz}$	Calc. $\Delta\delta_{trz}$
0	$4{,}94.10^{-3}$	0,00	0,00
$1{,}22.10^{-3}$	$4{,}89.10^{-3}$	0,09	0,09
$2{,}42.10^{-3}$	$4{,}85.10^{-3}$	0,17	0,17
$4{,}75.10^{-3}$	$4{,}75.10^{-3}$	0,28	0,28
$4{,}58.10^{-2}$	$4{,}58.10^{-3}$	0,35	0,36
$4{,}41.10^{-2}$	$4{,}41.10^{-3}$	0,38	0,37
$4{,}12.10^{-2}$	$4{,}12.10^{-3}$	0,38	0,38
$3{,}53.10^{-2}$	$3{,}53.10^{-3}$	0,38	0,39

K = 2000 M^{-1}, $\Delta\delta_{max}$ = 0.39 ppm.

Job plot experiments

Job plot of mono-triazolium with TBACl in $CDCl_3$

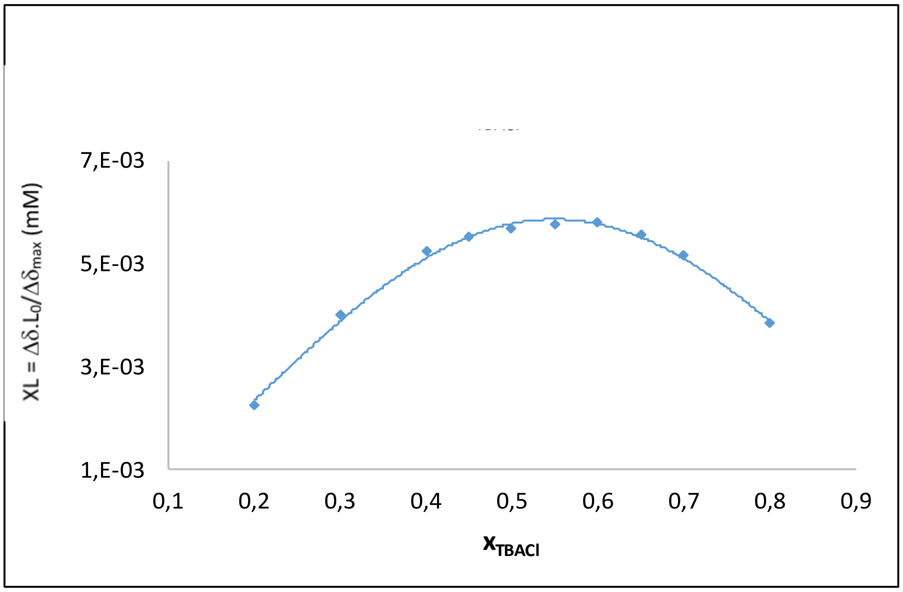

Job plot of mono-triazolium with $TBANO_3$ in $CDCl_3$

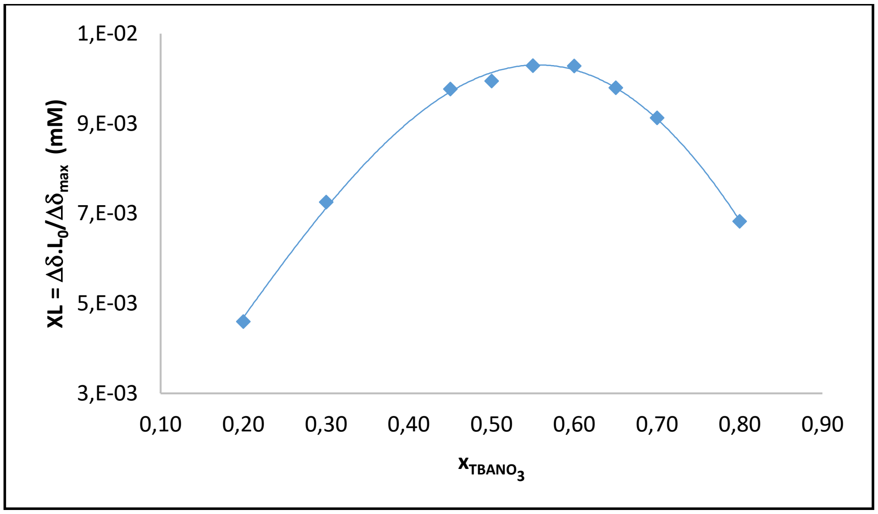

Job plot of bis-triazolium with TBACl in $CDCl_3$

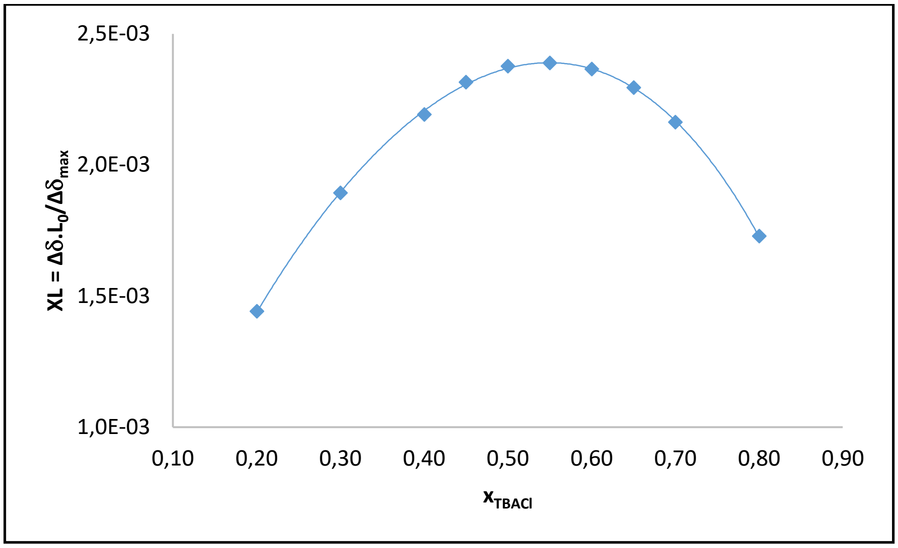

Job plot of bis-triazolium with $TBANO_3$ in $CDCl_3$

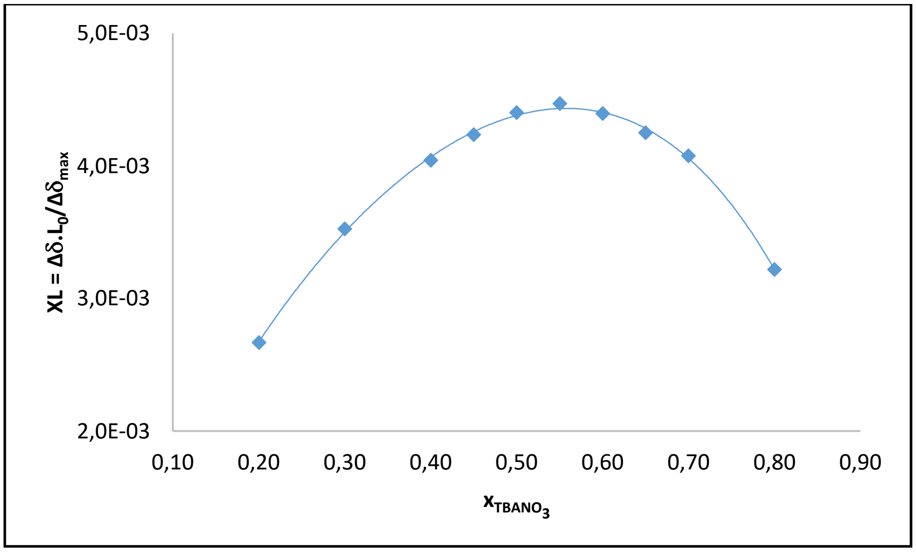

Computational details

Electronic energies were performed at the DFT level using the Gaussian 16. We used the M06-2X functional and a def2-TZVPP basis set with a continuum model of the chloroform. The integration grid was set to ultrafine. The Gibbs free energy was obtained using harmonic frequencies, rigid rotator and perfect gas approximation at a temperature of 298 K, but scaling down the entropic correction by 50% to take into account the change of reference from gas phase to solute and the reduction of translational entropy.

Table. Raw energetic data. Gibbs energy includes here 100% of the entropic contribution.

Name	Electronic Energy (Ha)	Electronic Energy + Zero Point Energy (Ha)	H (Ha)	G (Ha)
BF_4^-	-424.671956217	-424.657524	-424.65224	-424.685103
DMPT-1	-1288.7271457	-1288.314623	-1288.288273	-1288.372805
DMPT-1 , BF_4^-	-1713.43212469	-1713.003741	-1712.971391	-1713.070318
DMPT-2	-1328.4488514	-1327.99393	-1327.96607	-1328.05391
DMPT-2 , BF_4^-	-1753.173263	-1752.702342	-1752.668713	-1752.768154
DMPT-2 , BF_4^- , BF_4^-	-2177.877825	-2177.39178	-2177.351831	-2177.466728

Structures

DMPT-1

N -3.64758 -3.88335 -0.15321

N -3.87026 -2.79578 0.52184

C -2.75579 -2.06087 0.69689

C -1.75013 -2.76034 0.07287

N -2.37102 -3.86280 -0.42560

C -0.34192 -2.43015 -0.05108

N 0.02411 -1.16763 0.08287

C 1.40023 -1.16690 -0.01280

C 1.87464 -2.50325 -0.21801

C 0.77082 -3.30896 -0.24512

C 2.16792 0.00087 0.03703

C 1.61441 1.29536 0.07330

C 2.24605 2.54082 0.25787

C 1.26861 3.52139 0.24653

C 0.03880 2.87507 0.05699

N	0.26422	1.55642	-0.04288
C	-1.27655	3.46563	-0.03119
N	-1.46361	4.79904	0.15967
N	-2.71610	5.07031	0.01988
N	-3.35692	3.93820	-0.26151
C	-2.50395	2.90383	-0.30561
C	-4.79477	3.93524	-0.46559
C	-5.21640	-2.50265	1.00657
C	-1.79760	-4.95515	-1.20475
H	1.39443	4.58386	0.35851
H	3.30381	2.68290	0.39861
H	-5.12598	4.96782	-0.44420
H	-5.02544	3.49095	-1.42976
H	-5.27958	3.37390	0.32890
H	-2.82323	1.90149	-0.52905
C	3.64328	-0.11103	0.05230
H	-0.40778	0.79912	-0.11606
H	2.90159	-2.80167	-0.34622
H	-2.74125	-1.13660	1.24410
H	-5.20519	-2.50130	2.09221
H	0.75017	-4.37859	-0.36382
H	-5.51914	-1.53122	0.62850
H	-5.87342	-3.27925	0.63294
H	-1.12253	-4.53423	-1.94358
H	-1.26396	-5.63095	-0.54246
H	-2.61838	-5.47274	-1.68784
C	4.27695	-0.90395	1.01063
C	5.65945	-1.00443	1.03238
C	6.42241	-0.32886	0.08867
C	5.79877	0.45552	-0.87299

C	4.41731	0.57232	-0.88713
H	3.68092	-1.42365	1.74887
H	6.14100	-1.60980	1.78799
H	7.50049	-0.41298	0.10296
H	6.38808	0.97737	-1.61439
H	3.93030	1.17749	-1.64011

DMPT1, BF4

N	-3.01522	3.84853	-0.20629
N	-3.17075	2.80869	-0.96998
C	-2.03496	2.11007	-1.13000
C	-1.08298	2.78004	-0.39953
N	-1.75516	3.83047	0.13790
C	0.31568	2.44993	-0.19574
N	0.69532	1.20023	-0.37548
C	2.05197	1.18449	-0.14613
C	2.51088	2.50521	0.17439
C	1.40810	3.31237	0.15034
C	2.79515	0.00266	-0.13629
C	2.22018	-1.28096	-0.21188
C	2.86021	-2.52847	-0.35444
C	1.88627	-3.51035	-0.37865
C	0.64813	-2.86376	-0.25227
N	0.86349	-1.54200	-0.15851
C	-0.66792	-3.45671	-0.21991
N	-0.84753	-4.79657	-0.06527
N	-2.11312	-5.04814	-0.04757
N	-2.76371	-3.89421	-0.19159
C	-1.90984	-2.87041	-0.30961
C	-4.21415	-3.84763	-0.21807

C	-4.50279	2.46788	-1.45415
C	-1.25194	4.86659	1.02933
F	-1.80131	-0.07518	0.93619
B	-3.20186	0.00444	0.93023
F	-3.60342	1.27071	1.35770
F	-3.73934	-0.98844	1.73953
F	-3.65074	-0.19448	-0.39790
H	2.01565	-4.57342	-0.48077
H	3.92351	-2.66741	-0.45002
H	-4.58938	-4.64881	0.41064
H	-4.52925	-2.88404	0.17251
H	-4.57356	-3.97782	-1.23637
H	-2.23973	-1.85708	-0.44360
C	4.26825	0.08897	-0.01726
H	0.16860	-0.80561	-0.05190
H	3.52153	2.78686	0.41928
H	-1.97847	1.20319	-1.70075
H	-4.39152	1.85488	-2.34149
H	1.37410	4.37252	0.33591
H	-5.01028	1.90728	-0.67512
H	-5.02566	3.39001	-1.68357
H	-0.62706	4.39752	1.78353
H	-0.67825	5.59317	0.46019
H	-2.11069	5.34209	1.48928
C	4.99664	0.88552	-0.90229
C	6.37644	0.97042	-0.79598
C	7.04234	0.27494	0.20494
C	6.32408	-0.51210	1.09570
C	4.94588	-0.61196	0.98174
H	4.47500	1.42206	-1.68364

H	6.93188	1.57910	-1.49638
H	8.11810	0.34643	0.29092
H	6.83696	-1.04834	1.88240
H	4.38394	-1.21747	1.68024

DMPT2

C	4.31776	1.08743	-0.95409
C	3.69075	0.27316	-0.00813
C	4.46835	-0.40596	0.93174
C	5.84801	-0.27043	0.92603
C	6.46514	0.53029	-0.02580
C	5.69828	1.20473	-0.96786
C	2.22049	0.13040	0.00083
C	1.44931	1.33833	0.03303
C	1.87834	2.65175	0.23358
C	0.75198	3.47426	0.21682
C	-0.34833	2.64923	0.00497
N	0.08786	1.37330	-0.10583
C	-1.74670	2.97754	-0.16220
N	-2.36661	4.04819	0.40607
N	-3.62680	4.11802	0.08364
N	-3.84415	3.09719	-0.68801
C	-2.73693	2.35531	-0.88414
C	-5.17728	2.87044	-1.24240
C	1.65420	-1.12712	-0.01906
N	0.28685	-1.34916	0.09513
C	0.13134	-2.64757	-0.00416
C	1.37658	-3.34903	-0.19773
C	2.33373	-2.38661	-0.20442
C	-1.20265	-3.20988	0.14599

C	-2.29785	-2.68319	0.78558
N	-3.27552	-3.59650	0.63750
N	-2.88337	-4.63671	-0.03635
N	-1.63547	-4.40683	-0.33275
C	-4.63817	-3.54219	1.16290
C	-0.90494	-5.39860	-1.11846
H	0.73986	4.54526	0.31318
H	2.89781	2.96159	0.38273
H	-5.82039	3.66072	-0.87353
H	-5.11430	2.90277	-2.32564
H	-5.53255	1.90042	-0.90879
H	-2.72763	1.48634	-1.51683
H	-0.45382	0.51503	-0.15732
H	3.39307	-2.51986	-0.34459
H	-2.42745	-1.76384	1.32596
H	-4.60869	-3.72111	2.23358
H	1.52001	-4.41092	-0.29615
H	-5.04960	-2.56062	0.95244
H	-5.21225	-4.31336	0.66270
H	-0.32566	-4.88195	-1.87755
H	-0.25578	-5.97072	-0.46178
H	-1.64062	-6.04866	-1.57759
H	3.98425	-1.02034	1.67896
H	6.44034	-0.78895	1.66714
H	7.54181	0.63069	-0.03272
H	6.17603	1.82352	-1.71474
H	3.72154	1.60784	-1.69169
C	-1.80161	5.06495	1.28891
H	-2.62880	5.54020	1.80330
H	-1.13846	4.57541	1.99563

H	-1.25695	5.79442	0.69596

DMPT2, BF4

C	-4.91202	0.81597	0.90464
C	-4.29718	0.04068	-0.08065
C	-5.08977	-0.69050	-0.96686
C	-6.47233	-0.64289	-0.87160
C	-7.07622	0.12180	0.11762
C	-6.29372	0.84692	1.00767
C	-2.82334	-0.01988	-0.18257
C	-2.11978	1.22638	-0.27202
C	-2.64767	2.51168	-0.42801
C	-1.58290	3.41189	-0.44537
C	-0.42155	2.65976	-0.30483
N	-0.75676	1.35455	-0.20716
C	0.96581	3.07283	-0.30344
N	1.43896	4.20913	0.27438
N	2.73180	4.32238	0.15631
N	3.10999	3.26327	-0.49574
C	2.08177	2.45940	-0.81517
C	4.52264	3.05200	-0.79918
C	-2.19392	-1.24518	-0.17027
N	-0.82396	-1.38073	-0.33446
C	-0.56589	-2.64957	-0.14503
C	-1.75153	-3.42505	0.14242
C	-2.77672	-2.53541	0.12017
C	0.81413	-3.09570	-0.25989
C	1.85589	-2.50935	-0.93530
N	2.92537	-3.28367	-0.68782
N	2.64149	-4.29389	0.08088

N	1.36793	-4.18254	0.33707
C	4.30648	-3.05968	-1.10325
C	0.72972	-5.17526	1.19458
F	1.72889	0.04171	0.71843
B	3.12834	-0.09240	0.73664
F	3.58691	-0.13841	-0.60055
F	3.47754	-1.27455	1.38074
F	3.69495	1.00907	1.37042
H	-1.63604	4.47841	-0.57795
H	-3.69182	2.74784	-0.53638
H	5.10349	3.44441	0.02788
H	4.67544	1.98250	-0.89633
H	4.76747	3.57046	-1.72169
H	2.21419	1.53279	-1.34205
H	-0.12337	0.56719	-0.07606
H	-3.81784	-2.73230	0.31393
H	1.90366	-1.61796	-1.53128
H	4.29123	-2.52691	-2.04707
H	-1.81152	-4.48456	0.32400
H	4.79594	-2.46003	-0.34198
H	4.78387	-4.02708	-1.21214
H	0.09456	-4.65956	1.90830
H	0.14475	-5.85760	0.58445
H	1.51902	-5.71218	1.70788
H	-4.61597	-1.27919	-1.74114
H	-7.07745	-1.20249	-1.57151
H	-8.15442	0.15373	0.19482
H	-6.75996	1.43714	1.78451
H	-4.30243	1.37461	1.60223
C	0.69135	5.24223	0.98280

H	-0.07346	4.75937	1.58337
H	1.39367	5.77603	1.61251
H	0.23756	5.91915	0.26426

DMPT2 BF4 BF4

C	5.42077	0.84028	-0.70678
C	4.67984	0.10652	0.22136
C	5.34558	-0.56417	1.24820
C	6.72730	-0.49738	1.34732
C	7.45837	0.22690	0.41521
C	6.80291	0.89141	-0.61390
C	3.20609	0.02532	0.11906
C	2.47839	1.25946	0.07092
C	2.95874	2.56725	0.20276
C	1.87565	3.43576	0.06952
C	0.74970	2.64205	-0.13246
N	1.12575	1.34695	-0.12716
C	-0.64823	2.99419	-0.26203
N	-1.12615	4.08872	-0.90982
N	-2.42889	4.14338	-0.88887
N	-2.80348	3.08552	-0.23005
C	-1.76766	2.33816	0.18611
C	-4.22209	2.81233	-0.02020
C	2.59910	-1.20998	0.05867
N	1.22205	-1.35257	0.00848
C	0.99558	-2.62752	-0.16868
C	2.21443	-3.40442	-0.23219
C	3.22267	-2.50663	-0.08432
C	-0.38809	-3.06632	-0.26728
C	-1.52063	-2.45036	0.21007

N	-2.54081	-3.22096	-0.20188
N	-2.14585	-4.25394	-0.88944
N	-0.84535	-4.16139	-0.92614
C	-3.96868	-2.97582	-0.02083
C	-0.08240	-5.17753	-1.64012
F	-1.11394	0.04072	-1.49348
B	-2.49033	-0.08029	-1.74911
F	-3.17769	-0.16096	-0.52536
F	-2.72554	-1.24214	-2.48475
F	-2.93176	1.04473	-2.44704
H	1.89094	4.50907	0.14730
H	3.98170	2.84022	0.39497
H	-4.77839	3.41294	-0.73080
H	-4.37638	1.75206	-0.18989
H	-4.48128	3.07231	1.00153
H	-1.89069	1.42779	0.74699
H	0.52849	0.53246	-0.27143
H	4.28156	-2.70421	-0.09843
H	-1.65580	-1.55069	0.78660
H	-4.07936	-2.31996	0.83529
H	2.30222	-4.47054	-0.35610
H	-4.34291	-2.49094	-0.91746
H	-4.45551	-3.93132	0.14273
H	0.67341	-4.68407	-2.24370
H	0.38163	-5.85230	-0.92613
H	-0.77719	-5.71916	-2.27155
H	4.77218	-1.12077	1.97758
H	7.23196	-1.01022	2.15459
H	8.53619	0.27442	0.49038
H	7.36874	1.45000	-1.34687

H	4.91060	1.35161	-1.51222
C	-0.37399	5.13426	-1.59161
H	0.45125	4.67083	-2.12404
H	-1.04969	5.62291	-2.28414
H	0.00046	5.84757	-0.86256
B	-2.67129	-0.00285	2.87586
F	-3.43981	1.07615	2.42343
F	-2.45227	0.10759	4.24345
F	-3.32520	-1.20754	2.59057
F	-1.43348	0.00960	2.19595
